# Regulation of Placental Development and Its Impact on Fetal Growth—New Insights From Mouse Models

**DOI:** 10.3389/fendo.2018.00570

**Published:** 2018-09-27

**Authors:** Laura Woods, Vicente Perez-Garcia, Myriam Hemberger

**Affiliations:** ^1^Epigenetics Programme, The Babraham Institute, Cambridge, United Kingdom; ^2^Centre for Trophoblast Research, University of Cambridge, Cambridge, United Kingdom

**Keywords:** placenta, trophoblast, mouse models, IUGR, fetal growth restriction, DMDD

## Abstract

The placenta is the chief regulator of nutrient supply to the growing embryo during gestation. As such, adequate placental function is instrumental for developmental progression throughout intrauterine development. One of the most common complications during pregnancy is insufficient growth of the fetus, a problem termed intrauterine growth restriction (IUGR) that is most frequently rooted in a malfunctional placenta. Together with conventional gene targeting approaches, recent advances in screening mouse mutants for placental defects, combined with the ability to rapidly induce mutations *in vitro* and *in vivo* by CRISPR-Cas9 technology, has provided new insights into the contribution of the genome to normal placental development. Most importantly, these data have demonstrated that far more genes are required for normal placentation than previously appreciated. Here, we provide a summary of common types of placental defects in established mouse mutants, which will help us gain a better understanding of the genes impacting on human placentation. Based on a recent mouse mutant screen, we then provide examples on how these data can be mined to identify novel molecular hubs that may be critical for placental development. Given the close association between placental defects and abnormal cardiovascular and brain development, these functional nodes may also shed light onto the etiology of birth defects that co-occur with placental malformations. Taken together, recent insights into the regulation of mouse placental development have opened up new avenues for research that will promote the study of human pregnancy conditions, notably those based on defects in placentation that underlie the most common pregnancy pathologies such as IUGR and pre-eclampsia.

## Introduction

Intrauterine growth restriction (IUGR; also referred to as fetal growth restriction, FGR) is a common pregnancy complication, affecting around 3–8% of pregnancies worldwide (1–3). IUGR overlaps but is distinct from the more general condition known as small for gestational age (SGA), which is most commonly defined as weight below the 10th percentile for the gestational age. Whilst SGA babies are small but may be physiologically normal, IUGR is a pathological condition defined as the failure of a fetus to attain its full genetic growth potential. IUGR is a leading cause of stillbirth, prematurity, cerebral palsy and perinatal mortality (4–6). In the most severe cases, IUGR can lead to embryonic lethality and miscarriage during pregnancy. IUGR babies may have to be delivered prematurely, and therefore IUGR is also an indirect cause of preterm birth (7). Moreover, IUGR increases an infants' lifelong risk of adverse health outcomes including long-term poor neurological development, poor postnatal growth, and other childhood conditions, including serious and long-lasting immune deficiencies. Even well beyond infancy and childhood, IUGR is associated with a significant increase in the risk of cardiovascular disease, diabetes mellitus and hyperinsulinemia (8–10). Despite the distinction between IUGR and SGA, it is noteworthy that SGA infants are also affected by increased perinatal morbidity and mortality similar to IUGR, including neurodevelopmental disorders, stillbirth, and lifelong risk of adverse health outcomes (11).

IUGR is a complex and multifactorial disorder with a wide spectrum of potential causes. Some of these originate in specific genetic defects or congenital abnormalities in the embryo itself, or are a consequence of intrauterine infections. However, the majority of IUGR cases are caused by a failure of the placenta (10). The placenta is the extra-embryonic organ that only persists for the duration of pregnancy but that is absolutely essential for all intrauterine development. It has a number of essential functions such as anchoring the conceptus to the uterine wall, producing hormones to sustain pregnancy, inducing an immune-privileged environment and—as the perhaps most widely appreciated role—providing the embryo with sufficient amounts of nutrients and oxygen. Because of the pivotal role of the placenta in the etiology of IUGR, we here provide a brief overview of placental development, focussing for the most part on the mouse as the genetically most tractable model system to study early developmental processes at the molecular level. Specifically, we concentrate on fetal-specific effects of gene mutations. Interactions between placental trophoblast cells and maternal immune cells are also known to have an influence on growth trajectories of the fetus, but this aspect is outside the scope of this review and has been summarized elsewhere (12). Following this, we discuss novel insights into genetic causes of placental dysmorphologies. We will focus on recent findings that highlight the highly under-estimated number of genes contributing to placental development, and offer first approaches on how these may serve to identify novel molecular hubs that may be of key importance for placentation. These advances in the field harbor the prospect of enabling a better appreciation of the various causes of IUGR at the molecular and genetic level, which may lead to improved disease sub-classification, refined diagnosis and potentially to improved treatment options in the future.

## Brief overview of placental development

**Figure 1:**
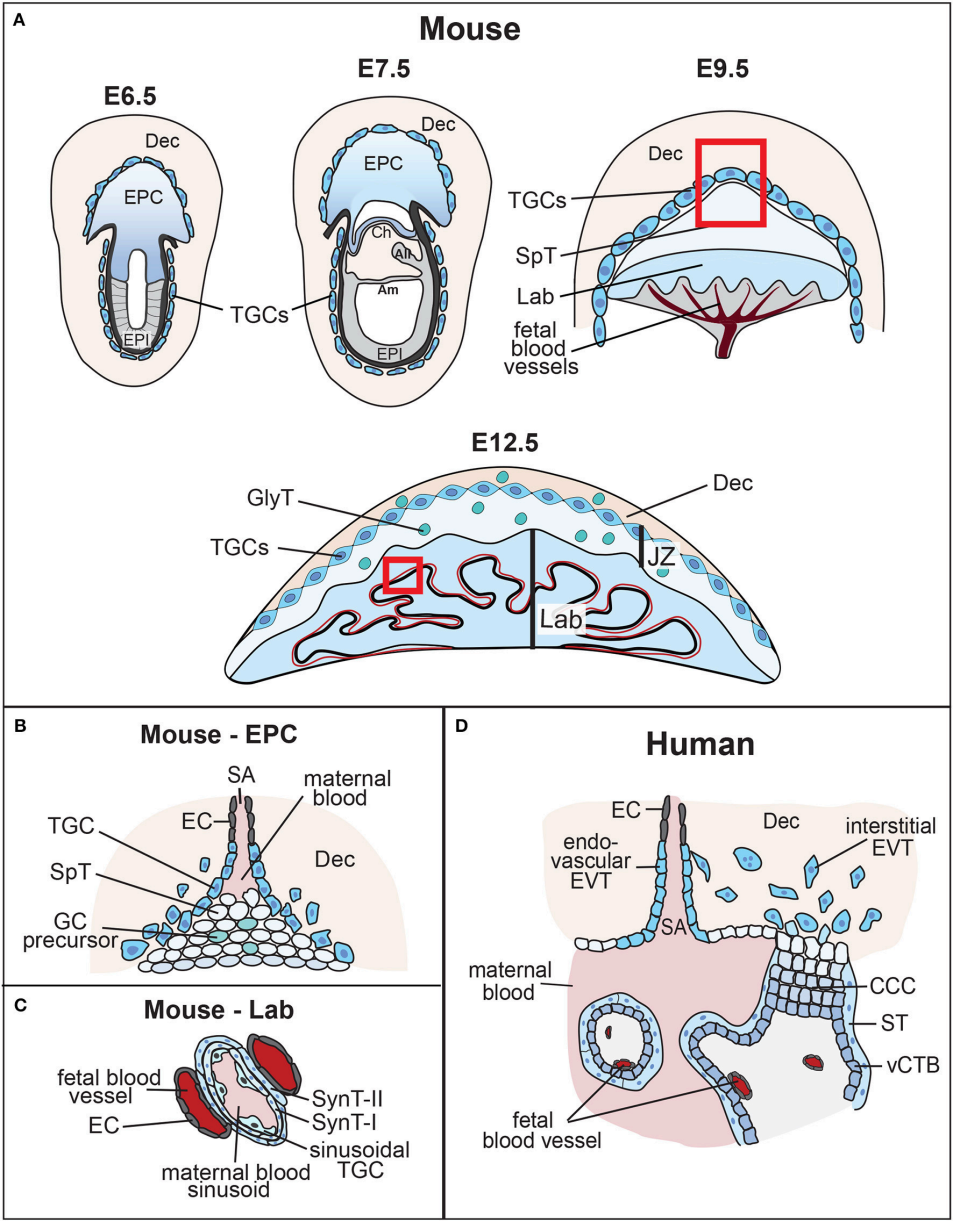
Overview of mouse placental development and similarities to human placenta. **(A)** Following implantation, the blastocyst's mural trophectoderm differentiates into trophoblast giant cells (TGCs), while trophectodermal cells that overlie the inner cell mass form the extra-embryonic ectoderm (ExE) and the ectoplacental cone (EPC). The future embryo originates from the blastocyst's inner cell mass that differentiates into the epiblast (EPI). The conceptus is embedded into the maternal decidua (Dec) which differentiates from the uterine endometrium. With gastrulation, the chorion (Ch) consisting of a trophoblast and an extra-embryonic mesodermal cell layer, the amnion (Am) and the allantois (All) are formed. Cells at the margins of the of the EPC differentiate into invasive, secondary TGCs, that remodel the maternal vasculature [highlighted by the inset, see **(B)**]. The allantois grows out and attaches to the chorion (chorio-allantoic fusion) around E8.5, a critical process for developmental progression. At E9.5, allantoic blood vessels start to invaginate into the chorionic ectoderm to initiate formation of the placental labyrinth (Lab). Trophoblast cells overlying this layer differentiate into future spongiotrophoblast (SpT) and glycogen cells (GCs). The mature mouse placenta is established around mid-gestation (~E10.5) and continues to grow in size and complexity. It consists of three main layers: the labyrinth (Lab), the junctional zone (JZ) made up of SpT, GCs and TGCs, and the maternal decidua (Dec). The labyrinth is the main site of nutrient and gas exchange [highlighted by the inset, see **(C)**]. Black bar indicates thickness of the labyrinth zone. **(B)** Magnified view of the tip of the EPC where invasive TGCs remodel maternal spiral arteries (SA) by eroding their smooth muscle lining and displacing their endothelial cell (EC) layer. **(C)** Close-up view of the interhaemal barrier, consisting (from maternal to fetal side) of a discontinuous layer of sinusoidal TGCs, two layers of syncytiotrophoblast (SynT-I and -II), and the endothelial cell layer of the fetal blood vessels. **(D)** Despite significant morphological differences, comparison with the human placenta reveals structural and/or functional similarities. Human placental villi are made up of a mesenchymal core that contains the fetal blood vessels, a layer of villous cytotrophoblast (vCTB) and one overlying layer of syncytiotrophoblast (ST) that is directly exposed to maternal blood. Thus, the haemochorial organization and the exchange barrier are similarly organized between mouse and human placentas. vCTBs are perhaps most analogous to the chorionic ectoderm in mice. In anchoring villi, cytotrophoblast cells form cytotrophoblast cell columns (CCCs) that invade into the maternal decidua (Dec). Cells at the base of the CCCs proliferate, pushing cells along the column where they progressively differentiate into invasive extravillous trophoblast (EVT). Trophoblast invasion occurs along two routes, interstitially into the decidual stroma, and along an endovascular route to replace the endothelial cell (EC) lining of maternal spiral arteries (SA). This spiral artery remodeling process is instrumental for healthy pregnancy progression, and is equally shared with the mouse where these processes occur between ~E7.5 and E10.5 as shown.

Tracing back in developmental time, the first definitive emergence of future placental cells occurs at the blastocyst stage with the formation of the trophectoderm. The trophectoderm forms the outer shell of the blastocyst, which is set aside from cells of the inner cell mass that will generate the embryo proper. Trophectoderm-derived cells ultimately give rise to all trophoblast cell types of the future placenta. Trophoblast cells make up the majority and defining aspects of the placenta. However, the most important exception to this placental cell provenance is the fetal placental vasculature. Endothelial cells of the fetal placental vasculature descend from cells of the extra-embryonic mesoderm that form the allantois and umbilical cord (13). These extra-embryonic mesodermal cells emerge slightly later in development at gastrulation, but ultimately have their cell lineage origin in the inner cell mass and epiblast. Thus, the placenta is a composite organ of two distinct cell lineages that arise from the fertilized embryo and that are established in early development, (1) the trophoblast lineage as the first lineage to differentiate that exclusively gives rise to placental trophoblast cell types, and (2) the extra-embryonic mesoderm that originates from cells of the inner cell mass and forms the fetal placental vasculature (Figure 1A).

Apart from these fetal-derived cell types, a vital aspect of placental development is the maternal uterine tissue into which the blastocyst is embedded upon implantation. The cells of the uterine lining, the so-called endometrium, undergo a specialized decidualization reaction upon implantation that is instrumental to support normal placentation and hence embryonic growth and survival (14). Indeed, the decidua will form part of the mature placenta. These principal tissue contributions are broadly similar between murine and human placentas. It is because of this complex and unique composition, combining fetal and maternal parts and thus amalgamating cells with different genetic constitution in a single organ, that often makes it difficult to discern where precisely the putative causes of a placental malformation may reside.

### Placentation in the mouse

Mouse models have been instrumental for advancing our knowledge of the impact of extra-embryonic development on pregnancy outcome and tracing the cell lineage origins of particular defects. For this reason, it is important to appreciate the progression of extra-embryonic development in the mouse, in comparison to that in humans. Following on from blastocyst formation, the trophectoderm cells surrounding the murine blastocoel cavity will soon cease to proliferate and enter endoreduplicative cell cycles, forming early (primary) trophoblast giant cells (TGCs). These TGCs help the embryo penetrate the uterine epithelium and implant into the endometrium, a process that takes place from developmental day (E) 4.5 onwards. Following implantation, polar trophectoderm cells overlying the inner cell mass continue to proliferate in response to a fibroblast growth factor 4 (FGF4) signal emanating from the epiblast (15). The arising column of diploid trophoblast cells forms the extra-embryonic ectoderm (ExE), a tightly packed epithelial tissue that retains stem cell potency (Figure 1A). Cells at the proximal tip of the ExE, farthest away from the epiblast, will further differentiate into the ectoplacental cone (EPC) that sits like a cap on top of the ExE. The cells at the margins of the EPC differentiate into secondary TGCs. It is these secondary TGCs in particular that acquire invasive characteristics, penetrating deeply into the endometrial stroma and making contact with maternal arteries. Trophoblast invasion by TGCs peaks between ~E7.5 and E9.5. This process entails that TGCs erode away the smooth muscle layer and displace the endothelial cell lining of maternal blood vessels (Figure 1B). As a consequence, maternal blood is funneled into the placenta in trophoblast-lined conduits and in the absence of vaso-constrictive control by the mother (16). These vascular remodeling processes are key to the successful progression of pregnancy, as they lay the anatomical foundations for the functional capacity of the developing placenta.

### Labyrinth formation

With gastrulation, cells of the ExE differentiate into the chorionic ectoderm. At the same time, the allantois develops from extra-embryonic mesoderm at the posterior end of the embryo, positioned exactly at the embryonic—extra-embryonic boundary. The allantois grows out and toward the chorion, ultimately attaching to it at around E8.5. This process of chorio-allantoic fusion is absolutely essential for further pregnancy progression. In its absence, development is abrogated soon afterwards. Chorio-allantoic fusion establishes a scenario where extra-embryonic mesodermal cells are in direct contact with the chorionic ectoderm, and start to invaginate into it in finger-like projections at sites pre-determined by expression of a particular transcription factor, *Gcm1* (17, 18). In the chorionic trophoblast, these invaginating mesodermal protrusions trigger a differentiation process in which individual trophoblast cells fuse to form syncytiotrophoblast. Syncytiotrophoblast cells ultimately will establish the transport surface, or “interhaemal membrane,” of the placenta (Figure 1C). They form the blood sinusoids through which maternal blood (brought in by the trophoblast-lined spiral arteries and canals) percolates, and across which nutrients and oxygen must be transported to reach the fetal blood circulation. In the mouse, the entire exchange barrier, from the maternal to the fetal side, is made up of a total of three continuous cell layers, two layers of syncytiotrophoblast (SynT-I and SynT-II, respectively) and the extra-embryonic mesoderm-derived fetal endothelial cells (19). Sinusoidal TGCs that are likely of chorionic trophoblast origin are also present at the maternal side, apposed to the SynT-I layer, but they only form a fenestrated, discontinuous layer that does not constitute a complete barrier (Figure 1C). These intricate developmental steps start to occur from around mid-gestation in the mouse (E9.5–10.5) and lead to the formation of the so-called labyrinth. With labyrinth formation, the mature mouse placenta is being established. The labyrinth continues to grow for the next days by continued branching morphogenesis leading to further elongation and refinement of these inter-digitated vascular spaces. This architecture achieves a large surface area for transport in which maternal and fetal blood circulations come into close contact but never mix. Moreover, maternal and fetal blood flow in a counter-current direction, thus optimizing transport capacity (20).

As can be appreciated from these complicated and intricate developmental processes, defects and deficiencies in labyrinth formation are a frequent cause of developmental failure and growth deficits, respectively. Up until mid-gestation, the yolk sac meets the nutritional needs of the early embryo. However, from around E10 onwards the transport capacity of the placenta is an absolute requirement to ensure embryo survival. Indeed, this requirement to switch from yolk sac nutrition to placental nutrient supply, tied to the necessity for chorio-allantoic fusion and labyrinth formation to occur successfully, creates a developmental bottleneck around mid-gestation in the mouse when a large proportion of mutants die.

### Junctional zone formation

The junctional zone (JZ) is positioned between the labyrinth and the maternal decidua. Together with the labyrinth, it forms the other major layer of the fetal part of the mature mouse placenta. The JZ originates mainly from cells of the core of the EPC, as judged by gene expression of prominent markers, such as *Tpbpa*. It contains three main cell types: spongiotrophoblast cells (SpT), glycogen cells (GCs) and a layer of TGCs that directly border the decidua (21). GCs often associate with maternal blood canals and sinuses. From about E12.5 onwards, they invade into the decidua where they become associated with maternal blood spaces. Because of their glycogen content and location, GCs are believed to serve as an energy store that can provide additional nutrition to the placenta and/or embryo. Apart from that, the JZ constitutes the main endocrine compartment of the placenta. It produces vast amounts of hormones, growth factors and cytokines that are important for the normal progression of pregnancy, acting on both the maternal and fetal physiology (22, 23).

### The human placenta

Although mammalian placentas are functionally convergent, placental morphology is remarkably different between species. Mouse and human placentas share in common a haemochorial type of placentation, meaning that fetal trophoblast cells are directly bathed in maternal blood. In both species trophoblast is invasive and penetrates deeply into the endometrium, in humans even farther reaching into the muscular layer of the myometrium. Cellular morphology and overlapping gene expression patterns have helped identify analogous cell types in mouse and human placentas, although a direct comparison is not always clear-cut. The structure analogous to the murine labyrinth are the placental villi in the human placenta. As in the mouse, the cells exposed to the maternal blood are syncytial in nature, however, in humans there is only one layer of syncytiotrophoblast (Figure 1D). Immediately underlying the syncytiotrophoblast is a layer of villous cytotrophoblast cells (vCTBs) that continuously fuse into the syncytium and replenish it. These cells may be analogous to chorionic ectoderm cells in the early mouse placenta, but whether such a cell population persists into later murine gestation remains unknown. The core of the villus is made up of mesenchymal cells, fetal blood vessels, and a macrophage cell type known as Hofbauer cells. The blood vessels come close to the vCTB layer at presumptive sites of nutrient transfer, which hence in humans has to cross at least one syncytiotrophoblast layer, one cytotrophoblast layer and the fetal endothelial cells (19).

While there is appreciable morphological and functional similarity between the mouse labyrinth and the human placental villous structure, the equivalent to the JZ is harder to make out. Conceivably, such a relationship exists with the cytotrophoblast cell columns (CCCs). These columns grow out at certain points from the villi and anchor the placenta to the uterine wall. They consist of tightly packed cytotrophoblast cells that are highly proliferative at the base of the CCC, thereby contributing to the growth of the column. At their distal tips, extravillous cytotrophoblast (EVT) cells leave the context of the column and invade deeply into the uterine stroma. As in the mouse, this process of trophoblast invasion is instrumental to remodel maternal spiral arteries into large, trophoblast-lined canals that bring blood into the placenta (24). From the base of the column to the tip, cytotrophoblast cells undergo a well-documented epithelial-mesenchymal transition that coincides with acquiring invasive characteristics (25, 26). Like TGCs, EVT cells are also becoming polyploid and/or aneuploid, but not to the same extent as in the mouse where the genome content of TGCs can gain an equivalent of up to 1000N. In many regards, this morphological layout is somewhat similar to the ExE-EPC structure in the early mouse conceptus. It translates less obviously into the JZ structure of the mature mouse placenta, although by cellular descent these cells will be related to their earlier ExE/EPC progenitors.

## Placental structures linked to embryonic growth

Despite this mixed picture of structural and functional similarities and discrepancies between mouse and human placentas, the mouse model has been instrumental for gaining insights into molecular pathways that direct early trophoblast cell fate decisions as well as for identifying genes that affect placental development. For this reason, we focus below on key examples of what we have learnt from mouse mutants over recent years.

### Maternal spiral arteries and blood canals

Trophoblast invasion and the remodeling of maternal spiral arteries are perhaps the most widely accepted processes associated with the patho-etiology of IUGR. A large number of genes have been linked to conferring invasive characteristics to mouse and human trophoblast, thereby endowing this cell type with the capacity to target and remodel maternal spiral arteries deep within the maternal decidua. Disruption of this process impairs placental blood flow and is a major cause of IUGR and pre-eclampsia (27). Pre-eclampsia is a pregnancy complication characterized by high blood pressure, proteinuria, and often fetal growth restriction. Both disorders share common risk factors and outcomes, however they are distinct conditions, i.e. not all cases of IUGR are also complicated by pre-eclampsia, and *vice versa*. In the mouse, invasive TGCs originate from the margins of the EPC. Their vital role in remodeling the maternal vasculature has been demonstrated in cell type-specific ablation experiments that took advantage of the regulatory elements of the *Tpbpa* gene to drive Cre recombinase expression. *Tpbpa* is a key marker gene of the precursors of invasive TGCs located within the core of the EPC. Ablation of *Tpbpa*-positive cells by conditional activation of a *Diphteria* toxin gene results in trophoblast invasion deficiencies and consequently in defective remodeling of maternal spiral arteries (28). Mature placentas from such mice exhibit a small JZ with reduced SpT, GC and TGC numbers, and the conceptuses die around E11.5. A comparable placental phenotype is observed upon deletion of the serine peptidase *Htra1*, which targets a similar cell population and partially ablates *Tpbpa-*positive EPC cells (29). These data show that in mouse, as in humans, trophoblast invasion and the vascular remodeling process mediated by these invading cells are pivotal determinants of fetal growth and survival.

In addition to these proof-of-concept phenotypes in the mouse, strong evidence suggests that the diameter of maternal blood canals is regulated by the NOTCH signaling pathway. Deletion of the transmembrane receptor *Notch2* in trophoblast causes developmental delay due to impaired invasion of maternal spiral arteries and a reduced size of maternal blood canals and -sinuses at the entry point into the placenta (30, 31). The importance of NOTCH signaling has also been demonstrated in the human placenta. In the first trimester placenta, NOTCH1 is expressed exclusively by progenitors of invasive EVTs (32), suggesting a role in spiral artery remodeling. In term placentas the NOTCH1,−2,−4 receptors, and the NOTCH pathway ligand JAGGED2 are expressed in the brush border of the syncytiotrophoblast layer, with NOTCH1 and NOTCH4 also marking vascular endothelial cells. Expression of these NOTCH family components is disrupted in placentas affected by IUGR and pregnancy-induced hypertension (33).

### Junctional zone defects

**Figure 2:**
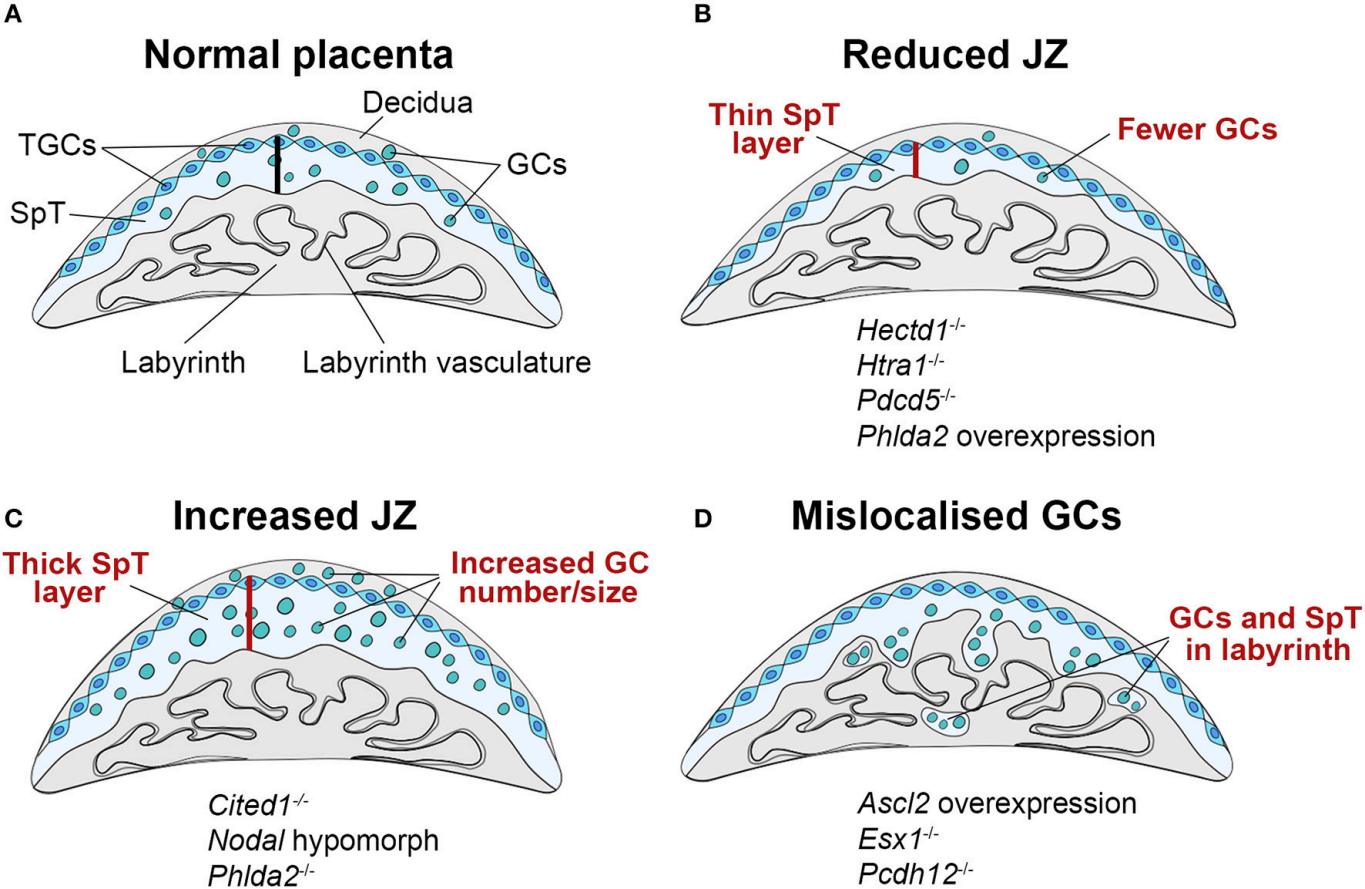
Schematic representation of JZ defects that can be associated with IUGR. **(A)** The junctional zone (JZ) is composed of three cell types: spongiotrophoblast cells (SpT), glycogen cells (GCs) and trophoblast giant cells (TGCs). The JZ provides energetic (glycogen), hormonal and physical support to ensure correct placentation and pregnancy progression. **(B–D)** Recurring JZ phenotypes entail **(B)** a reduced thickness of the JZ layer (indicated by the red bar) with fewer SpT and/or GCs, **(C)** an increased size of the JZ with more SpT and/or GCs and **(D)** a mislocalisation of GCs and SpT in the labyrinth. All of these phenotypes can be associated with IUGR, highlighting that JZ size alone is not indicative of placental efficiency, but that other parameters such as effect on cellular function, and precise cell localisation in relation to blood vessels and blood conduits, is important. Examples of mouse mutants or overexpression models in which these dysmorphologies are observed are given (fetal genotype is shown).

Numerous mouse models with JZ defects exhibit an IUGR phenotype (Figure 2, Supplementary Table 1). A small JZ is associated with impaired production of the large family of prolactin-like hormones. However, deletion of the gene encoding prolactin (*Prl*) alone does not cause overt JZ defects (34), potentially implying functional redundancy with the other 22 prolactin-like genes present in the mouse genome (35). Alternatively, the abnormal expression of prolactin family hormones that is observed in many mutants with JZ defects may represent a readout rather than a cause of abnormal JZ size. In any case, fetal growth is affected by the size of the developing JZ. Apparently, this effect is indeed linked to the JZ and not the placenta as a whole, as fetal growth is impaired in mouse models which exhibit differences in the JZ but not the labyrinth (36, 37). JZ defects cause an altered endocrine environment in the placenta locally, but also have systemic effects on both fetus and mother. Such dysmorphologies may therefore affect fetal growth due to deregulation of the control of placental growth and structure, or by controlling the allocation of maternal resources, for example by altering maternal insulin resistance (38).

A major component of the JZ are GCs. Their ability to store and release large amounts of glycogen suggests they play a role in supplying glycogen as an energy source to the growing conceptus. For example, the volume of the JZ, the proportion of GCs it contains, and their total glycogen content is reduced when pregnant females are subjected to chronic or acute dietary restriction (39–41). Imprinted genes, i.e. the small collection of genes that are expressed in a parent-of-origin dependent manner, are a chief regulator of JZ development and maternal resource allocation. Disruption of imprinted gene expression in the placenta can be associated with both, fetal growth restriction or overgrowth in mice and humans. Prominent examples in humans are Beckwith-Wiedemann syndrome which is associated with fetal overgrowth, and Silver-Russell syndrome characterized by growth restriction. Both syndromes are caused by imprinting errors on chromosome 11p15, notably a paternal and maternal duplication of the imprinting control region, respectively. In the mouse, imprinting defects are commonly associated with JZ defects affecting the size of this compartment and hence the number of SpT and GCs (42). For instance, increasing the gene dosage of the imprinted gene *Phlda2* (encoding a pleckstrin homology domain protein) by a single copy dramatically reduces the size of the JZ and the amount of stored glycogen by between 25 and 35% (43). The remaining GCs fail to migrate into the decidua in late gestation but persist in the JZ. Instead, *Tpbpa*-positive SpT cells and GCs become progressively mislocalized in the labyrinth compartment. Partial loss-of-function of another imprinted gene, *Ascl2*, which regulates *Phlda2* expression, similarly leads to depletion of SpT and ablation of GCs, and fetal growth restriction (44).

The common co-occurrence of a growth-retarded fetus with reduced GC numbers and less total glycogen content suggests that IUGR could be linked to the scarcity of glycogen in these placentas (Figure 2B). However, overabundance of glycogen is also associated with reduced fetal growth (Figures 2C,D). This is observed in the *Phlda2* knockout, which is associated with expansion of the junctional zone resulting in an over-accumulation of placental glycogen to 3x its normal levels, and leads to fetal growth restriction (45). Similarly, overexpression of *Ascl2* or knockout of *H19*, a maternally expressed non-coding RNA, leads to increased placental glycogen stores and reduced fetal growth (46, 47). An increase in GC numbers and glycogen content can also be associated with GC mislocalisation. This is the case in the *Ascl2* overexpression model or also in knockouts of the cell adhesion molecule *Pcdh12* or the homeobox gene *Esx1*, where GCs are ectopically located in the labyrinth (47–49). Hence, GC mislocalisation is emerging as a common feature of several gene manipulations that cause JZ defects associated with fetal growth restriction (Figure 2D). Although it may appear counter-intuitive that increased placental glycogen stores restrict fetal and placental growth, it may be that in such cases GCs are unable to supply their stored glycogen to the conceptus. This could be due to the inability of GCs to migrate into the decidua and access the maternal blood supply, or failure to break down and release stored glycogen, resulting in its over-accumulation. These deficits in turn may be caused by cell intrinsic defects in glycogen release, or altered hormonal control of glycogen metabolism, potentially due to defects in hormone production by the JZ. Taken together, these findings suggest that accumulation and storage of glycogen, and its subsequent release, are distinct and delicately balanced processes. Failure of GCs to either store adequate quantities of glycogen or to degrade and supply it as an energy source is detrimental to fetal and placental growth and development.

Although the human placenta does not contain specialized GCs akin to those found in the mouse, EVT cells located distally in the basal plate have the ability to store and metabolize large quantities of glycogen (19). The human placenta accumulates glycogen mainly in the first trimester, which then declines toward term, potentially contributing to the increased fetal and/or placental growth at this time (50, 51). Several studies have investigated glycogen content and metabolism in placentas complicated by IUGR, however as of yet no differences have been found in glycogen deposition (52, 53), or the expression and activity of enzymes involved in glycogen synthesis (54). The role of placental glycogen in normal human pregnancy and in pregnancy disorders is relatively understudied, however a recent review highlighted the association between altered glycogen deposition and metabolism in pathological pregnancy, including pre-eclampsia with IUGR, and diabetes (55). The strong association between altered placental glycogen stores and IUGR in the mouse prompts further research into understanding the role of placental glycogen in healthy and pathological pregnancies.

### Labyrinth defects

**Figure 3:**
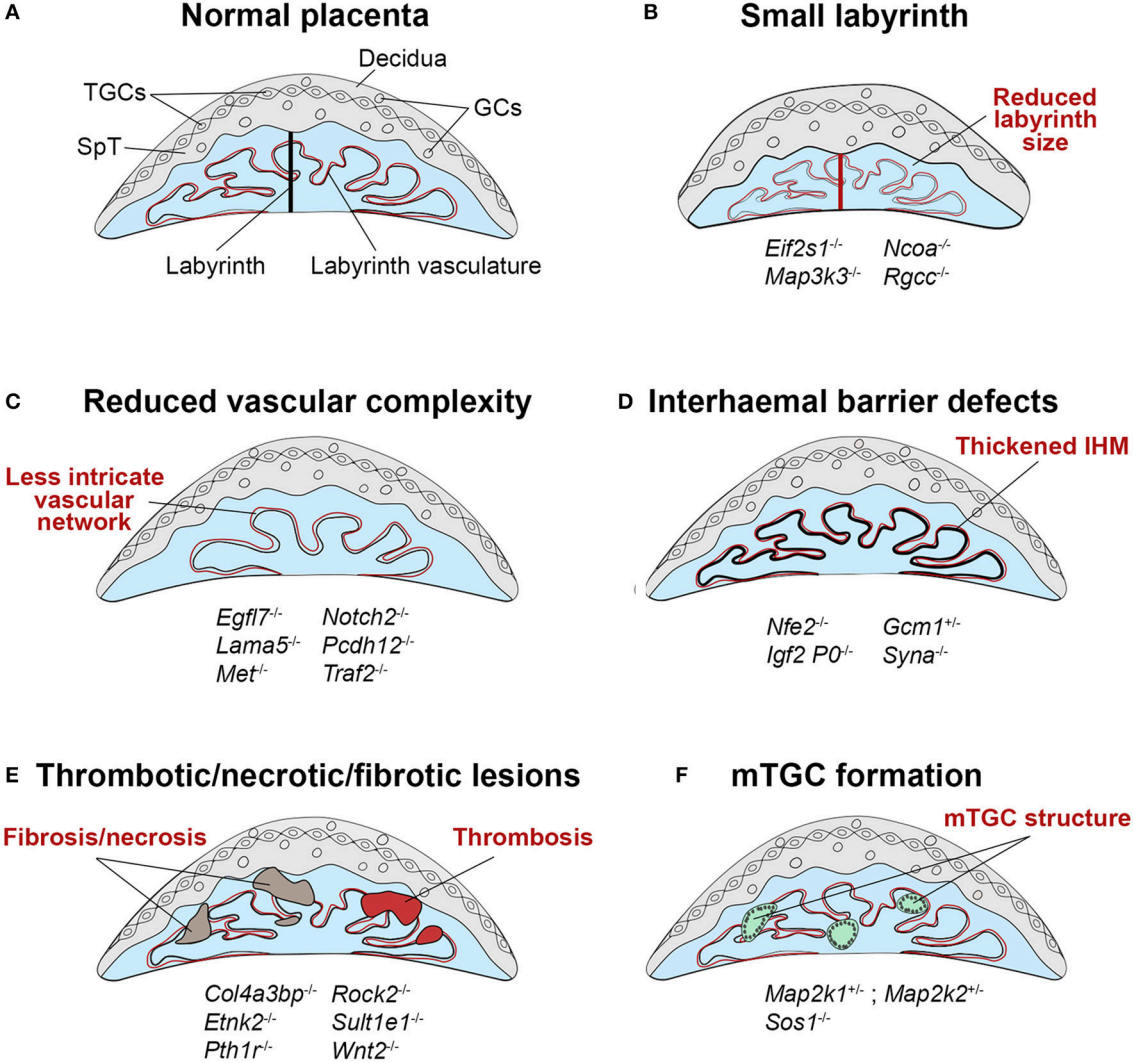
Schematic representation of labyrinth defects associated with IUGR. **(A)** Within a normal placenta, the labyrinth is the largest layer and is the site of all nutrient and gas exchange between the maternal and fetal blood circulations (black bar indicates thickness of the labyrinth layer). As such, failure in the establishment of this intricately organized layer is a direct cause of IUGR, or in more severe cases of intrauterine lethality. Defects in the development of the labyrinth often originate from defective or insufficient invagination of allantoic blood vessels into the chorionic ectoderm (see Figure 1, E9.5) and the subsequent branching morphogenesis that has to occur to form this exchange surface. **(B)** Such defects can lead to a small labyrinth layer (indicated by the red bar, often coupled with an overall reduced size of the placenta) or **(C)** a reduced complexity of the vascular organization within the labyrinth, in which there are fewer, disorganized and/or inappropriately dilated fetal blood vessels and maternal conduits. Principally, these structural defects lead to a reduction of the surface area available for transport, which hence cause placental insufficiency. **(D)** Impaired nutrient transfer may also occur because of a thickened or dysfunctional interhaemal barrier (IHM). **(E)** The transport surface may be diminished due to overt lesions in the labyrinth layer, that frequently entail thrombotic or necrotic patches or the accumulation of fibrotic tissue. **(F)** An unusual trophoblast differentiation defect is observed in some mouse mutants with the formation of multinucleate trophoblast giant cells (mTGCs). These mTGCs likely have their origin in inappropriate fusion and syncytialisation of labyrinth trophoblast cells, disrupting the intricate labyrinthine architecture. Examples of mouse mutants or overexpression models in which these dysmorphologies are observed are given (fetal genotype is shown).

The role of the labyrinth as the site of nutrient exchange strongly implicates this placental layer in the pathogenesis of IUGR. Any defect in the labyrinth which alters its ability to mediate the transfer of nutrients and oxygen to the fetus may impact on fetal growth. Such defects lead to an imbalance between the metabolic demands of the fetus and the ability of the placenta to meet that need. Indeed, in a recent systematic screen of embryonic lethal or sub-viable mouse mutants that are frequently associated with IUGR prior to death, labyrinth defects stood out as a prominent site of placental failure (56). Many other reports of individual gene knockouts support the direct link between placental labyrinth development and fetal growth and viability (Figure 3, Supplementary Table 1) (57).

#### Small labyrinth size

The size of the labyrinth compartment is an important determinant of its capacity to nourish the fetus. A small labyrinth has a reduced transport surface area, thereby limiting the nutrients available to the fetal circulation and hence restricting the extent of fetal growth (Figure 3B). A small labyrinth can be the result of a labyrinth-restricted failure to expand this particular compartment, or because of an overall reduced placental size. For example, deletion of the translation initiation factor *Eif2s1* causes endoplasmic reticulum stress in the placenta and specifically reduces the size of the labyrinth layer, resulting in growth restriction of the fetus (2). These scenarios can sometimes be difficult to distinguish, as the labyrinth forms a substantial fraction of the placenta, and hence labyrinth-to-JZ ratios are often used as a measure of disruptions in the relative proportion of labyrinth size. Labyrinth growth is regulated by genes encoding key growth factor pathway components, including the receptors for fibroblast growth factor (*Fgfr2*), leukemia inhibitory factor (*Lifr*), epidermal growth factor (*Egfr*) and hepatocyte growth factor (*Met*) (58). *Met* (also known as *c-Met*), for example, is involved in the proliferation of labyrinth trophoblast progenitors, and its deletion leads to reduced labyrinth size and cellularity, and poor development of the vascular branching structure (59). The *Fgfr2-IgIII*, and *Egfr* mutants are embryonic lethal at midgestation due to disrupted labyrinth and JZ development (60–63), whilst the *Lifr* mutant displays perinatal lethality with poor organization of the labyrinth and spongiotrophoblast (64).

The insulin-like growth factor (IGF) axis is another important regulator of placental growth and development, and the placenta itself is a major source of IGF2 during pregnancy (65). Deletion of the imprinted, placenta-specific *Igf2 P0* transcript significantly reduces labyrinth thickness and surface area (66, 67), causing fetal growth restriction. Human IUGR pregnancies are characterized by altered IGF signaling, with low levels of IGF1 in umbilical cord blood and increased IGFBP1 and IGFBP2 (68–70). Human *IGF1* mutations are associated with severe IUGR (71–73), suggesting the conserved importance of the IGF axis in placental development and fetal growth in mice and humans. The signaling cascades activated by the aforementioned growth factors and their receptors ultimately converge on the MAPK signaling cascade. Therefore, unsurprisingly, mutations in several MAPK pathway components also lead to varying degrees of labyrinth expansion deficits. As such, mutants of SOS1, GAB1, GRB2, RAF1, MEK1 (*Map2k1*), MEKK3 (*Map3k3*), and p38a (*Mapk14*) are characterized by a small labyrinth layer (Supplementary Table 1) (57, 74).

A small placental labyrinth is also a common feature of maternal undernutrition and diabetic mouse models leading to IUGR, showing that maternal exposure to external factors can affect labyrinth proliferation and expansion (39, 40, 75).

#### Vascular organization

The intricately branched network of fetal blood vessels in the labyrinth develops initially by vasculogenesis, when angioblasts that are specified in the allantois extend into the chorion to form the fetal labyrinth vasculature. As outlined above, failure in chorio-allantoic fusion prevents any such vascular development and is associated with mid-gestational lethality in the mouse. A key factor required for the specification of invagination points into the chorionic ectoderm is the transcription factor *Gcm1*. Homozygous *Gcm1* mutants develop normally until E9.5 but are growth restricted and die at E10.5 due to failing branching morphogenesis of the labyrinth. Instead, the chorion remains a flat layer in these mutants (17). Apart from complete failure of vessel invagination, milder forms of chorion folding defects and insufficient vessel invagination can also be observed in many other mutants (Figure 3C, Supplementary Table 1). Development of the chorio-allantoic vasculature is in part regulated by the TGF-β pathway, and *Tgfb, Alk1*, and *Alk5* null mutants all show labyrinth vascularisation defects (76, 77). Additionally, mutations in the WNT and BMP family components *Wnt2, Rspo3, Bmp4* and *Smad1* display abnormalities such as a small or absent allantois and failure of chorio-allantoic fusion, as well as defects in formation of the labyrinth vasculature, including failure of fetal vessels to invaginate into the chorion, and the inappropriate formation of large edematous maternal blood spaces (77). The PPAR signaling pathway also plays a role, as *Pparg* null trophoblast cells fail to undrego terminal syncytium formation, which disrupts the integrity of the vascular exchange interface and restricts growth of the fetus (78, 79). Members of the PPAR family, as well as *GCM1* and its antagonist *NFE2* have been implicated in human IUGR placentas (80–82), demonstrating that abnormal differentiation of the villous trophoblast, especially of the syncytiotrophoblast layer, also contributes to placental insufficiency and IUGR in humans.

From mid-gestation onwards, the fetal vessels undergo extensive branching by angiogenesis, which contributes to placental expansion to meet the increasing needs of the growing fetus. These processes are regulated by members of the vascular endothelial growth factor (VEGF), placental growth factor (PGF), FGF, WNT, and the TGF-β and BMP families (83). Disruption of such key angiogenic pathways in the placenta has significant impact on placental vascularisation and is causally associated with IUGR in mouse and human pregnancies (84). Commonly, placentas exhibiting such vascularisation defects show a reduction in the number of fetal and/or maternal blood spaces, or an abnormal lumen diameter (85–88). For example, mouse mutants with deletion of *Rgcc*, a VEGF-inducible angiogenic inhibitor (89), develop a small placenta with fewer and wider fetal capillaries. Poorly vascularised *Rgcc*^−/−^ placentas show decreased expression of *Vegf2* and *Pgf*, and embryos are growth restricted from E16.5 onwards (87).

The VEGF and PGF families constitute some of the prime regulators of angiogenesis in the labyrinth. Deletion of *Vegf* or overexpression of *Pgf* leads to severe labyrinth vascularisation defects, causing IUGR and embryonic death due to placental insufficiency (90–92). Similarly, ablation of the VEGF receptor *Vegfr1* (*Flt1*) in the mouse causes vasculogenesis and angiogenesis problems, resulting embryonic lethality (93). Overexpression of the soluble isoform of the human VEGF receptor, sFLT1, in mouse embryos induces pre-eclampsia like symptoms in pregnant dams, including IUGR, hypertension, and proteinuria, which can be ameliorated by PGF induction suggesting that PGF works antagonistically with sFLT1 to regulate placental angiogenesis (94). Dysregulated expression of sFLT1, PGF, and other angiogenic factors has been observed in human pregnancies complicated by IUGR and early onset pre-eclampsia complicated by IUGR (95–97). Indeed, determination of the sFLT1:PGF ratio, in combination with ultrasound findings, shows promise as a test of pre-eclampsia risk in the clinic, with an elevated ratio as an indicator of disease risk in pregnant women before the clinical onset of symptoms (98, 99).

#### Interhemal barrier defects

In addition to reduced labyrinth size, placental transfer insufficiency can also be caused by defects in the exchange barrier itself (Figure 3D). This may entail a mis-regulated expression of particular transporter molecules, or an increased thickness of the interhaemal membrane (IHM), thus hampering transport efficiency. These defects may occur in isolation or as compounding factor of small labyrinth size or reduced vascular complexity.

The IHM is a selectively permeable trilaminar barrier whose surface area increases throughout gestation as the labyrinth expands and becomes more intricately branched. The IHM also becomes significantly thinner between E12.5 and E16.5 of pregnancy, thereby reducing the distance over which substances must diffuse between the maternal and fetal blood supplies. This increases the theoretical diffusion capacity of the placenta, and in conjunction with expansion and branching of the labyrinth maximizes materno-fetal exchange (100, 101). Therefore, an increased thickness of the IHM can impede the efficiency of nutrient transfer to the fetus (Figure 3D). Disrupted morphology and thickness of the IHM is evident in the *Igf2 P0* knockout and in Syncytin A (*Syna*)-ablated mice, both of which display fetal growth impairment followed by embryonic lethality (67, 102). Manipulation of *Gcm1* expression to levels both below or above normal, by heterozygous *Gcm1* deletion or upregulation due to deletion of its antagonist p45NF-E2 (*Nfe2*), respectively, increases the thickness of the IHM (86, 103). Although fetal growth in *Gcm1*^+/−^ conceptuses is not affected, wild-type females carrying *Gcm1*^+/−^ conceptuses develop late gestational hypertension similar to what is seen in pre-eclampsia patients. Human placentas from IUGR pregnancies express reduced levels of *NFE2*, leading to upregulation of *GCM1* and excessive development of the syncytium (82), suggesting that similar molecular pathways operate in the establishment of the human placental IHM and may contribute to IUGR when disrupted.

In addition to IHM thickness, nutrient supply deficits may also be caused by mis-expression of specific transporter proteins. The *Igf2 P0* knockout affects in particular the system A amino acid transport system. Embryos overexpressing human *sFlt1*, which as mentioned earlier is implicated in early-onset pre-eclampsia with IUGR, develop a small labyrinth with reduced expression of the glucose diffusion channel *Connexin26* (*Cx26*). In contrast to their decreased expression of *Cx26, sFlt1* overexpressing embryos increase fatty acid and cholesterol transport by upregulation of *Cd36* and *Abca1* expression, respectively (104). Similarly, mice subjected to dietary restriction during pregnancy increase system A amino acid transporter activity leading to increased fetal amino acid accumulation (39). Amino acid and fatty acid/cholesterol uptake is increased in placentas with reduced ability to transport glucose, which may represent an attempt to compensate for an insufficient glucose supply. In fact, one study found that in normal pregnancies, the smallest placentas in a litter are more efficient in their nutrient transfer capacity to promote adequate fetal growth (105). Thus, up to a certain extent, small placentas may be able to compensate for reduced labyrinth size by increasing transport efficiency, but as labyrinth defects become more severe such compensatory mechanisms are insufficient to support normal embryonic growth.

#### Vascular lesions

Additionally, defects in the integrity of the labyrinth can lead to the formation of edematous regions and thrombosis, generating infarcts which may impair fetal growth by disrupting the flow of fetal and maternal blood (Figure 3E). Mouse strains and mutants affected by placental thrombosis and infarction often show reduced fetal growth (106–109). Similar defects are a common feature of placentas from human pregnancies complicated by IUGR (110, 111). Likewise, failing development and impaired perfusion of the labyrinth can lead to tissue fibrosis and necrosis, thus limiting the available healthy labyrinth tissue for nutrient and gas exchange (Figure 3E) (112–114). For example, *Traf2*^−/−^ (a regulator of NF-kappa-B and JNK) and *Col4a3bp*^−/−^ (a collagen binding protein) placentas exhibit regions of fibrosis and necrosis within the vascularised labyrinth compartment, and deletion of the parathyroid hormone receptor *Pth1r* causes disruption of the labyrinth vasculature due to the presence of spongiotrophoblast inclusions and abnormal accumulation of maternal blood in the labyrinth (56).

Additionally, several mouse mutants with deletion of MAPK pathway components, including *Map2k1, Map2k2*, and the Ras guanine nucleotide exchange factor *Sos1*, display an unusual defect characterized by the formation of large multinucleate structures in the labyrinth (Figure 3F) (115, 116). These are referred to as multinucleate TGCs (mTGCs) in the literature but are unlikely to be true TGCs and may instead be a result of aberrant fusion of SynT-I and SynT-II, as they display characteristics of both layers (116). Mutants exhibiting mTGC formation also show reduced embryo growth (117) suggesting their presence may impede placental nutrient transfer due to severe disruption of the labyrinth branching structure and interruption of the vascular network.

As already alluded to, the placental defects described do not necessarily occur in isolation, as labyrinth growth, villous branching, and angiogenesis are interlinked. For example, a labyrinth with a simple branching structure is likely also small, with a reduced network of fetal capillaries. A placenta with failure in SynT differentiation or placental vascularisation may be both small, and have a thickened IHM due to defects in formation of the syncytium or fetal capillaries (67, 86, 102, 103). Defects in labyrinth morphogenesis and integrity are likely to also be affected by the presence of vascular thrombosis and infarction (106, 107).

Moreover, defects in labyrinth formation often co-occur with abnormal development of the JZ, suggesting that development of the two layers is interlinked. For example, deletion of *Ascl2* reduces the size of the JZ layer with an absence of GCs, in addition to severe defects in labyrinth morphogenesis and vascularisation (44). Maternal dietary restriction during pregnancy initially reduces the volume and glycogen content of the JZ with no effect on labyrinth size at E16, however by E19 the labyrinth compartment is significantly reduced in these placentas (39). These observations raise the possibility that hormone secretion by the JZ influences labyrinth morphogenesis, and JZ glycogen stores may be utilized during late gestation to fuel expansion of the labyrinth. In fact, the glycogen content of the JZ decreases in late gestation as GCs are lysed, coinciding with expansion of the labyrinth compartment at this time (100, 118). If so, this may contribute to abnormal development of the labyrinth in placentas with JZ defects (119, 120).

## Endometrial causes of IUGR

Development of the placenta is dependent upon successful implantation of the blastocyst into the uterine wall, followed by decidualization of the surrounding uterine stroma. This is necessary to facilitate a controlled trophoblast invasion into and remodeling of the stromal compartment, key pillars for subsequent placental development. Implantation and decidualization are regulated by a series of highly orchestrated and synchronized interactions between a competent blastocyst and the receptive endometrium. In the mouse, implantation occurs on the evening of day 4 of pregnancy when the blastocyst becomes attached to the receptive endometrium and starts to embed itself into the uterine wall. Implantation of the embryo is a prerequisite for pregnancy progression without which development cannot occur (121–123). Disruption of implantation and decidualisation, or a delay in its timing can create a ripple effect through pregnancy, leading to poor placentation and abnormal growth and development of the embryo (124). Maternal deletion of the cytosolic phospholipase A2 (*Pla2g4a*), prostaglandin synthase 2 (*Ptgs2*), or lysophosphatidic acid receptor 3 (*Lpar3*), factors involved in the prostaglandin synthesis pathway, or of the homeobox gene *Msx1*, causes implantation to be deferred beyond the normal window (125–127). This leads to a spectrum of complications ranging from mild to severe growth restriction through to embryonic demise, which may be secondary to developmental abnormalities of the placenta. A similar outcome was observed when blastocysts developed to E4 were allowed to implant in an E5 uterus (125). This pathological deferred implantation is distinct from embryonic diapause when a blastocyst is retained dormant in the uterus for an extended period before resuming implantation (128). Thus, defects in implantation can have a knock-on effect on development of the placenta, with ramifications for fetal growth and development. Human studies suggest that deferred implantation may be a factor in pregnancies affected by growth restriction. Late implantation occurring >12 days after ovulation in human pregnancy is associated with early pregnancy loss (129). Additionally, pregnancies with a longer interval between ovulation and implantation have a smaller first trimester crown-rump length than those that implant earlier (130), which has been shown to be a predictor of low birth weight and IUGR (131–133).

Implantation stimulates uterine stromal cells to proliferate and differentiate in response to progesterone. Decidualized uterine stromal cells acquire a unique secretory phenotype, which facilitates deep trophoblast invasion and remodeling of the maternal uterine stroma to form the maternal portion of the placenta, the decidua. Inhibiting formation of the decidua by deletion of key genes such as progesterone receptor (*Pgr*), *Bmp2* or *Hoxa11* abrogates the maintenance of pregnancy (134–137), and impaired growth of decidual tissue limits the size of the placenta. As such, defects which attenuate the decidualization response lead to the development of a small placenta with limited capacity to nourish the fetus, such as in mice with uterine-specific deletion of the TGF-β family receptor *Bmpr2* (138). Decidualization and formation of the decidua is dependent on the action of progesterone in an estrogen-primed uterus. Uterine stromal cells in the receptive endometrium express high levels of *Pgr* and its co-receptors including *Stat3*, which renders them competent to undergo decidualisation. Mice with deletion of *Pgr* or *Stat3* in the uterus are infertile as their progesterone resistance leads to complete failure of decidualization (135, 139). In recent studies into placental development with advanced maternal age in mice, it was shown that PGR and pSTAT3 levels are reduced in the uterine stroma of aged females, thus decreasing the ability of the endometrium to respond to progesterone (140, 141). This causes a blunted and delayed decidualization response with reduced proliferation and differentiation of uterine stromal cells, thus retarding the development of the decidua by up to 2 days in aged pregnancies. Such maternal-age related defects in the decidua impact on development of the trophoblast compartment, such that placentas from aged pregnancies often exhibit a small, malformed labyrinth and increased numbers of TGCs due to abnormal and skewed trophoblast differentiation. This is likely the result of dysregulation of the reciprocal signaling between the abnormal decidua and the trophoblast compartments. Other studies have similarly shown that defective decidualization has a knock-on effect on development of the trophoblast. Decidualization defects stemming from the maternal uterus can lead to reduced thickness of the labyrinth and SpT layers, with defects in GC differentiation and localisation (142–144), and increased differentiation into the TGC lineage (138, 143–146). These defects are likely to have a significant impact on placental function and fetal growth. Most strikingly, transfer of embryos from aged females into a young uterine environment rescues the placentation defects as well as embryonic growth and development, thus unequivocally attributing the effect to the mother and not the fetus (141). This is as of yet an understudied area in human pregnancy. Whilst abnormal decidualization with advanced maternal age has not yet been shown in humans, older women are at increased risk of pregnancy complications including fetal growth restriction and pre-eclampsia. The findings in the mouse suggest that this may be a result of defective decidualization due to endometrial dysfunction.

Abnormal decidualization can be associated with shallow invasion and inadequate transformation of the maternal vasculature, which is thought to be a causative factor and defining feature of obstetric complications such as pre-eclampsia and fetal growth restriction. In the mouse, deletion of the PI3K antagonist *Pten* in the decidua causes the development of an abnormally thick decidual compartment, due to decreased levels of apoptosis. The persistence of a thick decidual layer impedes trophoblast invasion and delays the remodeling of maternal blood vessels. This presumably limits the maternal blood supply to the placenta in early gestation, thus restricting fetal growth (142). Expression of a dominant negative form of the gap junction protein connexin-43 (*Gja1*) increases the expression of angiogenic factors *Flt1* and *Vegfa*, thus altering decidual angiogenesis. As a consequence, maternal blood spaces are disorganized and abnormally dilated, thus decreasing the surface area available for exchange (147). Conversely, deletion of the TGF-β family receptor *Bmpr2* in the uterus impairs decidual angiogenesis, resulting in a hypo-vascularised, under-perfused decidua with a poor capacity to nourish the fetus. This leads to reduced fetal growth and a miscarriage-like phenotype with progressive hemorrhaging of the placenta (138). These examples highlight the maternal impact on placentation and fetal growth as a consequence.

## New insights into molecular pathways contributing to placental development

Based on all the genetic and histopathological evidence summarized in the examples outlined above, it is clear that the placenta plays a pivotal role in the etiology of IUGR. With this in mind, the insights from a recent consortium study termed “Deciphering the Mechanisms of Developmental Disorders” (DMDD) is of key importance (148). This initiative aimed at systematically examining the impact of embryonic lethal or sub-viable gene mutations on embryonic and placental development in the mouse. It revealed that the placenta is far more frequently affected in these mutants than previously thought. Indeed, almost 70% of the mutant mouse lines examined that do not produce viable offspring at weaning exhibit a placental phenotype, a number that far exceeds the ~10% placentation defects estimated on the basis of published data deposited in the Mouse Genome Informatics (MGI) database (56). Even though this study was conducted on mutants that do not produce viable offspring at the expected Mendelian ratios post-weaning, the vast majority of these mutants is also affected by IUGR prior to demise. Thus, these insights are highly valuable as it is conceivable that hypomorphic mutations in such genes may contribute to the spectrum of IUGR pathologies in viable offspring.

The key realization that most embryonic lethal or subviable mouse mutations will also suffer from placentation defects is of great impact. With some ~5000 genes causing embryonic lethality, and an estimated two-thirds of these being associated with placental defects, there are literally hundreds if not thousands of genes that remain unknown for their role in placental development. At least in some instances, these placentation defects will be causative of the developmental retardation and embryonic lethal phenotype. Even with the relatively limited number of genes analyzed as part of DMDD, it was possible to determine functional gene “hubs,” i.e., clusters of physically or functionally interacting factors that very likely play pivotal roles in normal placentation. One example highlighted in that study was the Polycomb group gene *L3mbtl2*. Further mining of the DMDD database also reveals, for instance, the glycosylphosphatidylinositol (GPI)-anchor biosynthesis pathway as an important player in formation of the early labyrinth. Thus, efforts to identify embryonic lethal and subviable mutants are of immense value to identify novel genes, and functional protein complexes, that are important for normal placental development and hence also likely involved in the etiology of IUGR.

### Data mining approaches

Apart from providing a first systematic survey of placental pathologies in embryonic lethal mouse mutants, another key advantage afforded by the DMDD study is the parallel analysis of both embryo and placenta for all mutant lines, allowing statistical co-association analyses of phenotype correlations. This analysis revealed a strong link between placental defects and heart, brain, and vascular abnormalities. These co-associations are not entirely unexpected, as a “heart-placenta axis” that describes an inter-dependence in the development of both organ systems has previously been suggested (78). However, this concept has so far been based on the analysis of very few, select genes only. The clear-cut nature of these developmental correlations in an entirely unbiased survey was hence compelling. They may indeed prove of great benefit for hypothesis-generating approaches to identify novel candidates for functional analysis in placental development. Thus, gene mutations that affect embryonic viability as well as heart, brain, or vascular development should enrich for factors that are also involved in normal placentation.

Underpinning the validity of this rationale on the above example of the GPI-anchor biosynthesis pathway, mutations in human *DPM1* and *PGAP2*, two pivotal genes implicated in placentation failure in the mouse, cause congenital disorders in which severe neurological dysmorphologies are a common feature (149, 150). Similarly, mutations in human *PIGL*, another placental regulator and GPI anchor synthesis protein identified by the DMDD study, are linked to a general developmental delay causing congenital heart disease, ocular colobomas, ichthyosiform dermatosis, mental retardation, ear anomalies and epilepsy (CHIME) syndrome (151). The fact that deletion of these genes in the mouse induces a severe trophoblast phenotype suggests an essential role of the GPI pathway in regulating early placentation linked to heart and neural development in the fetus.

**Figure 4:**
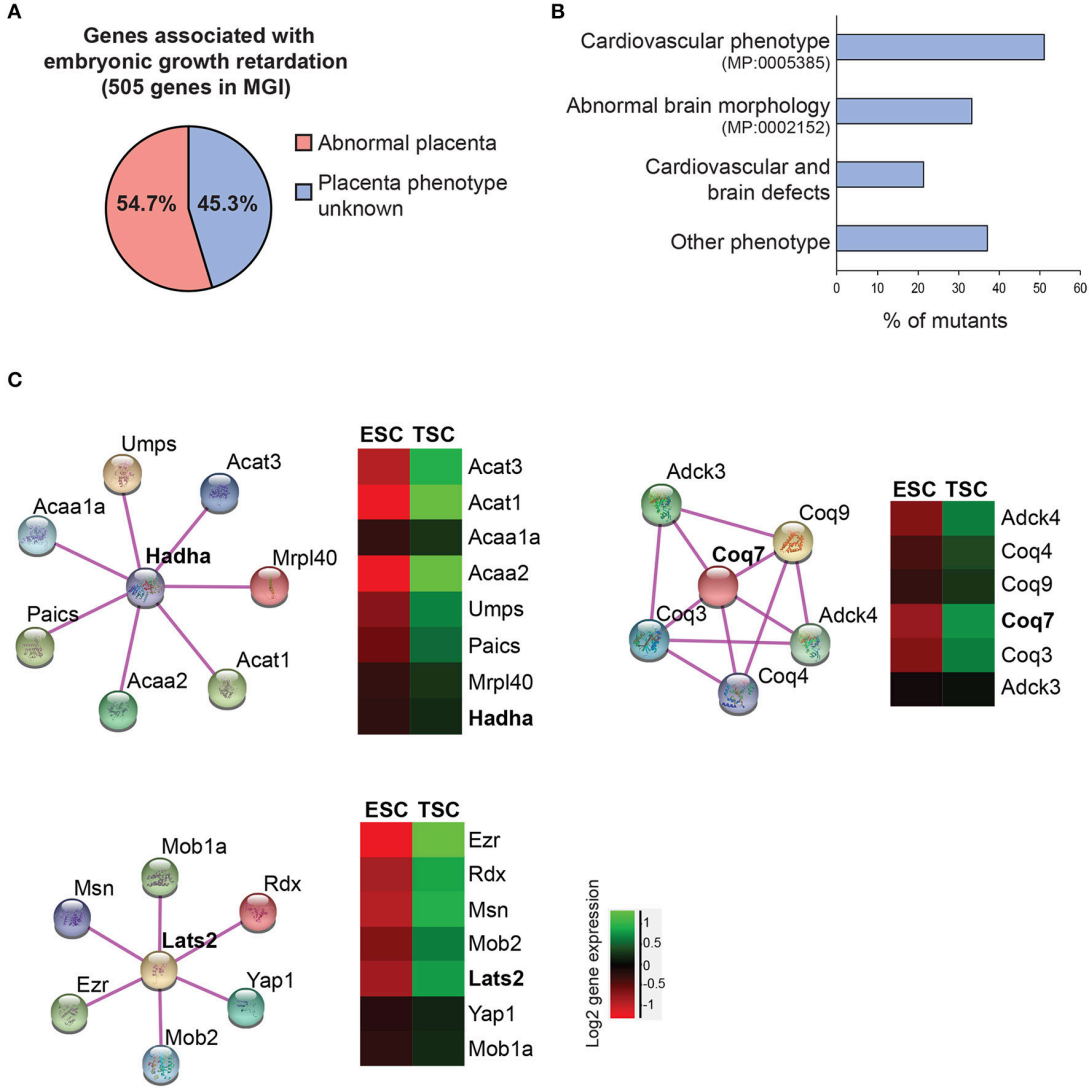
Data mining approaches to identify molecular networks of potential importance in placental development. **(A)** Pie chart of genes extracted from the Mouse Genome Informatics database (www.informatics.jax.org) that are associated with embryonic growth retardation (MP: 0003984; 505 genes in total) and that are either scored as having an abnormal placenta (MP:0002086 or MP:0004264) or an unknown placental phenotype. **(B)** Breakdown of the proportion of mutants with unknown placental phenotype that also exhibit defects in heart and/or brain development. Since placental defects are often co-associated with cardiovascular and brain abnormalities (56), this selection will enrich for genes that have a function in the placenta. **(C)** Molecular network analysis using String (https://string-db.org/) to identify physical and/or functional gene interactions for selected examples, and expression levels of the identified complex components in trophoblast stem cells (TSCs) compared to embryonic stem cells (ESCs). The network components are enriched in the trophoblast compartment, arguing for a potential function in placental development.

Following this line of thought, we here screened the MGI database for the mammalian phenotype term “embryonic growth retardation” (MP: 0003984). This revealed 505 gene mutations, of which 54.7% (276) are also known to exhibit placental abnormalities (Figure 4A). Intriguingly, the remaining 229 lines in which a placental phenotype remains unknown show a significant prevalence of cardiovascular system phenotypes (51.1%), brain development defects (33.2%), or both (21.4 %) (Figure 4B). Therefore, it is tempting to speculate that at least some of these gene mutations will also display placentation defects.

To take this analysis even further we screened the genes associated with cardiovascular and/or brain phenotypes for expression in embryonic (ESCs) and trophoblast (TSCs) stem cells. In doing so we uncovered several genes that cluster into functional networks with an enriched expression in the trophoblast compartment compared to ESCs, thus suggesting a likely role in placentation (Figure 4C). One such node, identified within the cardiovascular phenotype gene collection, centers around a hydroxyacyl-CoA dehydrogenase complex (*Hadha*) gene. *Hadha* encodes the alpha subunit of the mitochondrial trifunctional protein complex involved in the mitochondrial beta-oxidation of fatty acids. The gene, as well as its known interactors, shows increased expression in TSCs compared to ESCs, reinforcing the notion of the likely functionality of this complex in trophoblast differentiation. Along similar lines, the *Coq7* gene encoding the enzyme demethoxyubiquinone monooxygenase was identified in the overlap between search terms “embryonic growth retardation” and “abnormal brain morphology.” COQ7 is essential for ubiquinone biosynthesis. Ubiquinone acts as a cofactor during several redox processes like mitochondrial respiration. *Coq7*-deficient mice are embryonic lethal during mid-gestation with impaired neurogenesis and mitochondrial defects (152, 153). Although placental defects have not been described to date, *Coq7* is highly expressed in TSCs and placental trophoblast. Recently, a mutation of *COQ7* has been reported in a pregnancy complicated by oligohydramniosis, fetal lung hypoplasia, and growth retardation (154).

Finally, the joint overlap between search terms embryonic growth retardation, abnormal brain morphology and heart morphology reveals a number of genes that are highly expressed in TSCs, for example the muscle segment homeobox 2 (*Msx2*) transcription factor. *Msx1/2*-deficient mice show severe defects in cardiovascular and brain development and are growth-retarded (155). Our data would suggest that this mutant is likely to also exhibit defects in placental development. In fact, recent reports demonstrated that MSX2 is expressed in the human placenta and may regulate human trophoblast invasion (156). Another tangible example is the Hippo pathway component centered around *Lats2* (Figure 4C), that is known for its function in establishing cell polarity in the early embryo and the acquisition of trophoblast cell fate (15, 157). The phosphorylation state of YAP1 in particular is known as the broker between “inner” and “outer” cells in the morula-stage embryo, with unphosphorylated nuclear Yap1 being essential to induce trophoblast identity. The cytoskeletal protein Ezrin (*Ezr*) is a core hallmark of microvilli in epithelial cells and is strongly expressed in trophectoderm and its derivatives.

These various examples highlight the value of mining available large-scale datasets to identify novel candidate factors and pathways that may be required for normal placentation. Once identified, these putative placental hubs require in-depth phenotypic and functional screening to confirm their involvement in extra-embryonic development. Nevertheless, they may prove powerful to accelerate the much-needed efforts in gaining a broader understanding of the collection of genes involved in placental biology both in healthy and pathological conditions.

## Conclusions and outlook

Perhaps the most striking outcome of the recent DMDD mouse phenotyping screen is the realization of the extent to which the number of genes contributing to placental development has been underestimated. The above examples provide a starting point on how these new and extensive datasets can be mined in the future with the aim of identifying novel molecular networks that may have key functions during placentation. Even within the small set of genes highlighted here, it is clear that some have already been linked to human placentation and/or developmental problems. These links inspire with confidence that the combination of mouse phenotyping data, gene expression profiles, physical, and/or functional protein interaction networks and human disease links are indeed powerful approaches to identify such functional gene clusters. Arguably, rather than individual genes, the functionality of larger protein complexes and/or gene networks is more likely to be conserved between species, making this approach more powerful and more promising for translational studies. Once confirmed for an involvement in the etiology of placental defects, it is conceivable that such genes may become part of mutation screening panels for refined diagnosis and may constitute targets for drug development to improve our understanding and potential treatment options of IUGR in the future. Overall, the depth and molecular detail of the analysis of recent mouse mutants has opened up many new avenues for research aimed at understanding the molecular basis of normal placentation and of placental pathologies in humans.

## Author contributions

LW, VP-G, and MH contributed to the conceptualisation and writing of the manuscript. MH assembled Figure 1, LW Figures 2, 3, and VP-G Figure 4 and Supplementary Table 1.

### Conflict of interest statement

The authors declare that the research was conducted in the absence of any commercial or financial relationships that could be construed as a potential conflict of interest.
