# Supplementary material for: Regulation of Placental Development and Its Impact on Fetal Growth—New Insights From Mouse Models
Source: Front Endocrinol (Lausanne). 2018 Sep 27;9:570. doi: 10.3389/fendo.2018.00570 (PMC6170611; doi:10.3389/fendo.2018.00570)
Supplement: Supplementary file 1 [file Table_1.docx]

**Supplementary Table 1:** Placental defects in mouse mutants exhibiting varying degrees of IUGR

| **Gene** | **Gene product** | **Mutation** | **Lethality** | **Placental phenotype in mutant mice** | **Mouse reference** | **Involved in human IUGR** | **Human reference** |
| --- | --- | --- | --- | --- | --- | --- | --- |
| **Spongiotrophoblast defects** | | | | | | | |
| *Ascl2* | Achaete-scute homolog 2 | Targeted gene ablation | E9.5-E10.5 | Reduced SpT; increased TGC numbers | (Guillemot et al., 1994) |  |  |
| *Ascl2* | Achaete-scute homolog 2 | Hypomorph | IUGR (viable) | Small/absent JZ; fewer SpT and GCs | (Oh-McGinnis et al., 2011) |  |  |
| *Ascl2* | Achaete-scute homolog 2 | Over-expression | IUGR (viable) | Small JZ; increased GC numbers and mislocalisation | (Tunster et al., 2016b) |  |  |
| *Cited1* | Cbp/P300-Interacting Transactivator, With Glu/Asp-Rich Carboxy-Terminal | Targeted gene ablation | IUGR E18.5, perinatal lethality | Enlarged JZ, smaller labyrinth; enlarged maternal blood sinusoids | (Rodriguez et al., 2004) |  |  |
| *Egfr* | Epidermal growth factor receptor | Targeted gene ablation | E11.5-P20, depending on genetic background | Small JZ | (Sibilia and Wagner, 1995; Threadgill et al., 1995) |  |  |
| *Esx1* | Extraembryonic, spermatogenesis, homeobox 1 | Targeted gene ablation | IUGR (viable) | Enlarged JZ; increased SpT and GC numbers and mislocalisation | (Li and Behringer, 1998) | Yes | (Murthi et al., 2006) |
| *Hectd1* | HECT domain E3 ubiquitin protein ligase 1 | Deletion and kinase dead mutation | E13.5-E18.5 | Small JZ; fewer SpT and GCs | (Sarkar et al., 2014) |  |  |
| *Htra1* | High temperature requirement A1 serine peptidase 1 | Targeted gene ablation | Viable | Small JZ; fewer SpT and GCs | (Hasan et al., 2015) |  |  |
| *Nodal* | Nodal growth differentiation factor | Targeted gene ablation | E10.5 | Reduced SpT; increased TGC numbers | (Ma et al., 2001) |  |  |
| *Nodal* | Nodal growth differentiation factor | Hypomorph | E10.5 | Enlarged JZ and TGC layers | (Ma et al., 2001) |  |  |
| *Pdcd5* | Programmed cell death 5 | Targeted gene ablation | E13.5 | Small JZ; fewer GCs and TGCs; small labyrinth | (Li et al., 2017) |  |  |
| *Phlda2* | Pleckstrin Homology Like Domain Family A member 2 | Over-expression | IUGR (viable) | Small JZ; fewer GCs | (Tunster et al., 2010; Tunster et al., 2016a) |  |  |
| *Phlda2* | Pleckstrin Homology Like Domain Family A member 2 | Targeted gene ablation | IUGR (viable) | Enlarged JZ; increased GC numbers | (Tunster et al., 2016a) | Yes | (McMinn et al., 2006; Kumar et al., 2012) |
| *Pcdh12* | Protocadherin 12 | Targeted gene ablation | IUGR (viable) | Increased GC numbers and mislocalisation | (Rampon et al., 2008) |  |  |
| *Tfap2c* | Transcription factor AP-2 gamma | Deletion in *Tpbpa*+ cells | IUGR (viable) | Small JZ | (Sharma et al., 2016) |  |  |
| **Labyrinth defects** | | | | | | | |
| *Akt1* | Thymoma viral proto-oncogene 1; PKBa | Targeted gene ablation | IUGR (viable) | Small labyrinth; vascularisation defects | (Yang et al., 2003) | Yes | (Laviola et al., 2005) |
| *Arnt* | Aryl hydrocarbon receptor nuclear translocator; Hif1-β | Targeted gene ablation | E9.5-E10.5 | Small labyrinth; failure to initiate chorio-allantoic branching morphogenesis | (Kozak et al., 1997; Adelman et al., 2000) |  |  |
| *Birc6* | Baculoviral IAP repeat-containing 6; Bruce | Targeted gene ablation | Perinatal lethality | Labyrinth normal size; decreased vascular branching, small JZ | (Lotz et al., 2004) |  |  |
| *Ccnf* | Cyclin F | Targeted gene ablation | E10.5 | Small labyrinth; chorio-allantoic fusion failure, lack of fetal labyrinthine vasculature | (Tetzlaff et al., 2004a) |  |  |
| *Chm* | Choroideremia (RAB escort protein 1) | Targeted gene ablation | E11.5 | Small placenta; very small/absent labyrinth | (Shi et al., 2004) |  |  |
| *Cited1* | Cbp/P300-Interacting Transactivator, With Glu/Asp-Rich Carboxy-Terminal | Targeted gene ablation | Growth-restricted E18.5, perinatal death | Enlarged JZ, smaller labyrinth; enlarged maternal blood sinusoids | (Rodriguez et al., 2004) |  |  |
| *Col4a3bp* | Collagen, type IV, alpha 3 (Goodpasture antigen) binding protein | Targeted gene ablation | P14 subviable | Vascular lesions in labyrinth, fibrotic and/or necrotic  patches | (Perez-Garcia et al., 2018) |  |  |
| *Ctbp2* | C-terminal binding protein 2 | Targeted gene ablation | E10.5 | Small labyrinth; reduced chorio-allantoic branching and vascularisation | (Hildebrand and Soriano, 2002) |  |  |
| *Cyr61* | Cysteine rich protein 61 (ECM protein and integrin ligand); Ccn1 | Targeted gene ablation | E11.5 – P1 | Small labyrinth; chorio-allantoic fusion failure and branching morphogenesis defect | (Mo et al., 2002) |  |  |
| *Dlx3* | Distal-less homeobox 3 | Targeted gene ablation | E9.5- E10.5 | Small labyrinth | (Morasso et al., 1999) | Yes | (Chui et al., 2011) |
| *Eif2s1* | Eukaryotic Translation Initiation factor 2, subunit 1 Alpha | Gain-of-function | Perinatal lethality | Small labyrinth | (Yung et al., 2012) |  |  |
| *Egfl7* | Epidermal growth factor-like domain 7; vascular endothelial-statin | Targeted gene ablation | IUGR (viable) | Small labyrinth; reduced fetal vascularisation | (Lacko et al., 2017) |  |  |
| *Egfr* | Epidermal growth factor receptor | Targeted gene ablation | E12.5 – P21 depending on genetic background | Small labyrinth | (Sibilia and Wagner, 1995; Threadgill et al., 1995) | Yes | (Fondacci et al., 1994) |
| *Esx1* | Extraembryonic, spermatogenesis, homeobox 1 | Targeted gene ablation | IUGR (viable) | Placental hyperplasia, interhaemal barrier and vascularisation defects | (Li and Behringer, 1998) | Yes | (Murthi et al., 2006) |
| *Etnk2* | Ethanolamine kinase 2 | Targeted gene ablation | Perinatal death | Extensive placental thrombosis | (Tian et al., 2006) |  |  |
| *Fbxw7* | F-box and WD-40 domain protein 7; Fbw7 | Targeted gene ablation | E10.5-E13.5 | Small labyrinth; failure to initiate chorio-allantoic branching morphogenesis | (Tetzlaff et al., 2004b) |  |  |
| *Fgfr2* | Fibroblast growth factor receptor 2 | Targeted gene ablation | E10-E11 | Small/absent labyrinth; failure of chorio-allantoic attachment | (Arman et al., 1998; Xu et al., 1998) |  |  |
| *Flt1* | FMS-like tyrosine kinase 1; sFLT | Overexpression | IUGR (viable) | Small labyrinth and loss of GCs | (Kuhnel et al., 2017) | Yes | (Nevo et al., 2008) |
| *Flt1* | FMS-like tyrosine kinase 1; Vegfr1 | Targeted gene ablation | E8.5-E9.5 | Small labyrinth; vascularisation defects | (Fong et al., 1995) | Yes | (Sibley et al., 2002) |
| *Fosl* | Fos-like antigen 1; Fra1 (AP-1 transcription factor component) | Targeted gene ablation | E10.0 and E10.5 | Small labyrinth; chorio-allantoic branching morphogenesis defect | (Schreiber et al., 2000) |  |  |
| *Gab1* | Growth factor receptor bound protein 2-associated protein 1 | Targeted gene ablation | E16.5-E17.5 | Small labyrinth | (Itoh et al., 2000) |  |  |
| *Gcm1* | Glial cell missing homolog 1 | Targeted gene ablation | E10.5 | Small labyrinth; failure to initiate chorio-allantoic branching morphogenesis | (Anson-Cartwright et al., 2000) | Yes | (Baczyk et al., 2009; Kohli et al., 2017) |
| *Gcm1* | Glial cell missing homolog 1 | Hypomorph (heterozygous deletion) | IUGR (Viable) | Small labyrinth; Interhaemal membrane defects | (Bainbridge et al., 2012) |  |  |
| *Gjb2* | Gap junction protein, beta 2; Connexin 26 | Targeted gene ablation | E11 | Small labyrinth; vascularisation defects and nutrient transfer impairment | (Gabriel et al., 1998) |  |  |
| *Gjc1* | Gap junction protein, gamma 1; Connexin 45 | Targeted gene ablation | E9.5-E10.5 | Small labyrinth; vascularisation defects | (Kruger et al., 2000) |  |  |
| *Grb2* | Growth factor receptor bound protein 2 | Hypomorhic mutation | Perinatal lethality | Small labyrinth | (Saxton et al., 2001) |  |  |
| *Hectd1* | HECT domain E3 ubiquitin protein ligase 1 | Null mutation and kinase dead mutation | E13.5-E18.5 | Small labyrinth; fetal vascularisation defects, hemorrhages | (Sarkar et al., 2014; Sarkar et al., 2016) |  |  |
| *Hgf* | Hepatocyte growth factor | Targeted gene ablation | E14.5-E16.5 | Small labyrinth | (Schmidt et al., 1995; Uehara et al., 1995) | Yes | (Somerset et al., 1998) |
| *Igf2 (P0)* | Insulin-like growth factor 2 | Deletion of trophoblast-specific P0 transcript | IUGR (viable) | Small placenta; reduced labyrinth thickness | (Constância et al., 2002; Sibley et al., 2004) | Yes | (McMinn et al., 2006) |
| *Itga4* | Integrin alpha 4 | Targeted gene ablation | E10.5  (50 % penetrance) | Small labyrinth | (Yang et al., 1995) |  |  |
| *Itgav* | Integrin alpha V (fibronectin receptor alpha) | Targeted gene ablation | E9.5-E11.5 (80% penetrance) | Small labyrinth; reduced fetal vascularisation | (Bader et al., 1998) |  |  |
| *Itgb8* | Integrin beta 8 | Targeted gene ablation | E11.5 (60% penetrance) | Small labyrinth; reduced thickness and vascularisation defects | (Zhu et al., 2002) |  |  |
| *Junb* | Jun B proto-oncogene (AP-1 transcription factor component) | Targeted gene ablation | E10.0 | Small labyrinth; vascularisation defects | (Schorpp-Kistner et al., 1999) | Yes | (Rajaraman et al., 2010) |
| *Krt8/Krt19* | Keratin8/keratin19 | Compound deletion | E10 | Lack of labyrinth, increased number of TGCs | (Tamai et al., 2000) |  |  |
| *Lama5* | Laminin alpha 5 | Targeted gene ablation | E17 | Small labyrinth; reduced branching of blood vessels | (Miner et al., 1998) |  |  |
| *Lifr* | Leukemia inhibitory factor receptor | Targeted gene ablation | Perinatal lethality | Small labyrinth; vascular lesions | (Ware et al., 1995) |  |  |
| *Mapk1* | Mitogen-activated protein kinase 1; Erk2 | Targeted gene ablation | E11.5 | Small labyrinth | (Hatano et al., 2003) | Yes | (Laviola et al., 2005) |
| *Map2k1* | Mitogen-activated protein kinase kinase 1; Mek1 | Targeted gene ablation | E10.5 | Small labyrinth; vascularisation defects | (Giroux et al., 1999; Bissonauth et al., 2006) |  |  |
| *Map2k1+/-; Map2k2+/-* | Mitogen-activated protein kinase kinase 1/2 | Double heterozygous deletion | ~P14 | Multinucleate trophoblast giant cells in labyrinth (mTGCs) | (Nadeau and Charron, 2014) |  |  |
| *Map3k3* | Mitogen-activated protein kinase kinase 3; Mekk3 | Targeted gene ablation | E11 | Small labyrinth; fetal vascularisation defects | (Yang et al., 2000) |  |  |
| *Mapk7* | Mitogen-activated protein kinase 7; Erk5 | Targeted gene ablation | E10.5-11.5 | Labyrinth vascularisation defects, reduced diameter of fetal vessels | (Sohn et al., 2002) |  |  |
| *Mapk14* | Mitogen-activated protein kinase 14; p38a | Targeted gene ablation | E10.5 -E12.5 | Small labyrinth | (Adams et al., 2000; Mudgett et al., 2000) | Yes | (Laviola et al., 2005) |
| *Met* | Met proto-oncogene, tyrosine-protein kinase | Targeted gene ablation | E14.5-E16.5 | Small labyrinth; vascularisation defects and reduced cellularity | (Ueno et al., 2013) |  |  |
| *Nfe2* | Nuclear factor, erythroid derived 2 | Targeted gene ablation | IUGR (viable) | Increased IHM thickness, vascularisation defects | (Kashif et al., 2011) | Yes | (Kohli et al., 2017) |
| *Nfe2l2* | Nuclear factor, erythroid derived 2, like 2; Nrf2 | Targeted gene ablation | Viable | Small labyrinth | (Kweider et al., 2017) |  |  |
| *Ncoa6* | Nuclear receptor coactivator 6 (peroxisome proliferator-activated receptor-interacting protein) | Targeted gene ablation | E11.5-12.5 | Small labyrinth; chorio-allantoic branching morphogenesis defect | (Kuang et al., 2002) |  |  |
| *Notch1/ Notch4* | Notch1/Notch4 | Compound deletion | E9.5 | Small labyrinth; failure to initiate chorio-allantoic branching morphogenesis | (Krebs et al., 2000) | Yes | (Sahin et al., 2011) |
| *Notch2* | Notch 2 | Targeted gene ablation | E11.5 | Labyrinth vascularisation defects; reduced maternal blood sinus formation | (Hamada et al., 2007) | Yes | (Sahin et al., 2011) |
| *Pcdh12* | Protocadherin 12 | Targeted gene ablation | IUGR (viable) | Labyrinth vascularisation defects, reduced cellular density | (Rampon et al., 2008) |  |  |
| *Pdgfb* | Platelet-derived growth factor | Targeted gene ablation | Perinatal lethality | Small labyrinth; less trophoblast, dilated fetal blood vessels | (Ohlsson et al., 1999) |  |  |
| *Pnpla6* | Patatin-like phospholipase domain containing 6; Nte | Targeted gene ablation | E9.5 | Small labyrinth; chorio-allantoic branching morphogenesis defect | (Moser et al., 2004) |  |  |
| *Pgf* | Placental growth factor | Overexpression in T cells | IUGR (viable) | Small labyrinth; vascularisation defects | (Kang et al., 2014) | Yes | (Arroyo and Winn, 2008) |
| *Plk2* | Polo-like kinase 2; Snk | Targeted gene ablation | IUGR (viable) | Small labyrinth; decreased cell proliferation | (Ma et al., 2003) |  |  |
| *Pparg* | Peroxisome proliferator-activated receptor gamma | Targeted gene ablation | E9.5-E10 | Small labyrinth, decreased vascularisation | (Barak et al., 1999; Parast et al., 2009) | Yes | (Holdsworth-Carson et al., 2009; Holdsworth-Carson et al., 2010) |
| *Pth1r* | Parathyroid hormone 1 receptor | Targeted gene ablation | P14 lethal | Vascular lesions in labyrinth with (maternal blood) thrombosis; SpT and GC mislocalisation in labyrinth | (Perez-Garcia et al., 2018) |  |  |
| *Raf1* | V-raf-leukemia viral oncogene 1 | Targeted gene ablation | E9.5-P0 | Small labyrinth; vascularisation defects and reduced cellularity; small JZ | (Huser et al., 2001) |  |  |
| *Rb* | Retinoblastoma tumor suppressor | Targeted gene ablation | E13.5 | Excessive trophoblast proliferation in labyrinth; vascular lesions | (Wu et al., 2003) | Yes | (Rajaraman et al., 2010) |
| *Rbpj* | Recombination signal binding protein for immunoglobulin kappa J region; Rbpsuh | Targeted gene ablation | E9.5-10.5 | Small labyrinth and JZ; failure to initiate chorio-allantoic branching morphogenesis | (Krebs et al., 2004) |  |  |
| *Rgcc* | Regulator of cell cycle; Rgc32 | Targeted gene ablation | IUGR (viable) | Small labyrinth;  vascularisation defects | (Cui et al., 2013) |  |  |
| *Rock2* | Rho-associated kinase 2; signaling from Rho to actin skeleton | Targeted gene ablation | E13.5-E14.5 | Disruption of labyrinth architecture, thrombus formation | (Thumkeo et al., 2003) |  |  |
| *Rspo3* | R-spondin 3 | Targeted gene ablation | E11.5 – P0 | Small labyrinth; chorio-allantoic branching morphogenesis defect | (Aoki et al., 2007) |  |  |
| *Sos1* | SOS Ras/Rac guanine nucleotide exchange factor 1 | Targeted gene ablation | E9-E11 | Small labyrinth; multinucleate trophoblast giant cells in labyrinth | (Qian et al., 2000) |  |  |
| *Stk11* | Serine/threonine kinase 11; Lkb1 | Targeted gene ablation | E11 | Small labyrinth; failure to initiate chorio-allantoic branching morphogenesis | (Ylikorkala et al., 2001) |  |  |
| *Sult1e1* | Sulfotransferase family 1E, member 1 | Targeted gene ablation | IUGR (viable) | Placental thrombosis and degeneration | (Tong et al., 2005) |  |  |
| *Syna* | Syncytin A | Targeted gene ablation | E11.5-E13.5 | Labyrinth vascularisation defects; failure to establish IHM due to trophoblast fusion defects | (Dupressoir et al., 2009) |  |  |
| *Synb* | Syncytin B | Targeted gene ablation | IUGR (viable) | Vascularisation defects, enlarged maternal blood sinuses | (Dupressoir et al., 2011) |  |  |
| *Tfeb* | Transcription factor EB | Targeted gene ablation | E9.5-10.5 | Small labyrinth; Reduced vascularisation | (Steingrimsson et al., 1998) |  |  |
| *Traf2* | TNF receptor-associated factor 2 | Targeted gene ablation | P14 lethal | Small labyrinth; Reduced vascularisation, fibrotic/necrotic patches | (Perez-Garcia et al., 2018) |  |  |
| *Ube2l3* | Ubiquitin-conjugating enzyme E2L 3; UbcM4 | Inactivation by proviral integration | E11.5 | Small labyrinth; vascularisation defects, increased branching and variable vessel diameters | (Harbers et al., 1996) |  |  |
| *Ubp1* | Upstream binding protein 1; *LBP-1a* | Targeted gene ablation | E10.5 | Small labyrinth; reduced branching of blood vessels | (Parekh et al., 2004) |  |  |
| *Vcam1* | Vascular cell adhesion molecule 1 | Targeted gene ablation | E11.5-E12.5 | Small Labyrinth; chorio-allantoic fusion failure | (Gurtner et al., 1995; Kwee et al., 1995) | Yes | (Zygmunt et al., 1997) |
| *Vegfa* | Vascular endothelial growth factor A | Targeted gene ablation | E9.5 | Small labyrinth; vascularisation defects | (Carmeliet et al., 1996; Ferrara et al., 1996) | Yes | (Lyall et al., 1997; Szentpeteri et al., 2013) |
| *Wnt2* | Wingless homologue; secreted glycoprotein | Targeted gene ablation | Perinatal lethality (partial) | Small labyrinth; vascularisation defects, fibrotic lesions | (Monkley et al., 1996) | Yes | (Xiao et al., 2016) |
| *Zfp36l1* | Zinc finger protein 36, C3H type-like 1 | Targeted gene ablation | E11 | Small labyrinth; chorio-allantoic branching morphogenesis defect | (Stumpo et al., 2004) |  |  |

**SUPPLEMENTARY REFRENCES**

Adams RH, Porras A, Alonso G, Jones M, Vintersten K, Panelli S, et al. Essential role of p38alpha MAP kinase in placental but not embryonic cardiovascular development. *Mol Cell* (2000) 6:109-116.

Adelman DM, Gertsenstein M, Nagy A, Simon MC, and Maltepe E. Placental cell fates are regulated in vivo by HIF-mediated hypoxia responses. *Genes Dev* (2000) 14:3191-3203.

Anson-Cartwright L, Dawson K, Holmyard D, Fisher SJ, Lazzarini RA, and Cross JC. The glial cells missing-1 protein is essential for branching morphogenesis in the chorioallantoic placenta. *Nat Genet* (2000) 25:311-314. doi: 10.1038/77076.

Aoki M, Mieda M, Ikeda T, Hamada Y, Nakamura H, and Okamoto H. R-spondin3 is required for mouse placental development. *Dev Biol* (2007) 301:218-226. doi: 10.1016/j.ydbio.2006.08.018.

Arman E, Haffner-Krausz R, Chen Y, Heath JK, and Lonai P. Targeted disruption of fibroblast growth factor (FGF) receptor 2 suggests a role for FGF signaling in pregastrulation mammalian development. *Proc Natl Acad Sci U S A* (1998) 95:5082-5087.

Arroyo JA, and Winn VD. Vasculogenesis and angiogenesis in the IUGR placenta. *Semin Perinatol* (2008) 32:172-177. doi: 10.1053/j.semperi.2008.02.006.

Baczyk D, Drewlo S, Proctor L, Dunk C, Lye S, and Kingdom J. Glial cell missing-1 transcription factor is required for the differentiation of the human trophoblast. *Cell Death Differ* (2009) 16:719-727. doi: 10.1038/cdd.2009.1.

Bader BL, Rayburn H, Crowley D, and Hynes RO. Extensive vasculogenesis, angiogenesis, and organogenesis precede lethality in mice lacking all alpha v integrins. *Cell* (1998) 95:507-519.

Bainbridge SA, Minhas A, Whiteley KJ, Qu D, Sled JG, Kingdom JC, et al. Effects of reduced Gcm1 expression on trophoblast morphology, fetoplacental vascularity, and pregnancy outcomes in mice. *Hypertension* (2012) 59:732-739. doi: 10.1161/HYPERTENSIONAHA.111.183939.

Barak Y, Nelson MC, Ong ES, Jones YZ, Ruiz-Lozano P, Chien KR, et al. PPAR gamma is required for placental, cardiac, and adipose tissue development. *Mol Cell* (1999) 4:585-595.

Bissonauth V, Roy S, Gravel M, Guillemette S, and Charron J. Requirement for Map2k1 (Mek1) in extra-embryonic ectoderm during placentogenesis. *Development* (2006) 133:3429-3440. doi: 10.1242/dev.02526.

Carmeliet P, Ferreira V, Breier G, Pollefeyt S, Kieckens L, Gertsenstein M, et al. Abnormal blood vessel development and lethality in embryos lacking a single VEGF allele. *Nature* (1996) 380:435-439.

Chui A, Evseenko DA, Brennecke SP, Keelan JA, Kalionis B, and Murthi P. Homeobox gene Distal-less 3 (DLX3) is a regulator of villous cytotrophoblast differentiation. *Placenta* (2011) 32:745-751. doi: 10.1016/j.placenta.2011.07.007.

Constância M, Hemberger M, Hughes J, Dean W, Ferguson-Smith A, Fundele R, et al. Placental-specific IGF-II is a major modulator of placental and fetal growth. *Nature* (2002) 417:945-948.

Cui XB, Guo X, and Chen SY. Response gene to complement 32 deficiency causes impaired placental angiogenesis in mice. *Cardiovasc Res* (2013) 99:632-639. doi: 10.1093/cvr/cvt121.

Dupressoir A, Vernochet C, Bawa O, Harper F, Pierron G, Opolon P, et al. Syncytin-A knockout mice demonstrate the critical role in placentation of a fusogenic, endogenous retrovirus-derived, envelope gene. *Proc Natl Acad Sci U S A* (2009) 106:12127-12132. doi: 10.1073/pnas.0902925106.

Dupressoir A, Vernochet C, Harper F, Guegan J, Dessen P, Pierron G, et al. A pair of co-opted retroviral envelope syncytin genes is required for formation of the two-layered murine placental syncytiotrophoblast. *Proc Natl Acad Sci U S A* (2011) 108:E1164-1173. doi: 10.1073/pnas.1112304108.

Ferrara N, Carver-Moore K, Chen H, Dowd M, Lu L, O'Shea KS, et al. Heterozygous embryonic lethality induced by targeted inactivation of the VEGF gene. *Nature* (1996) 380:439-442.

Fondacci C, Alsat E, Gabriel R, Blot P, Nessmann C, and Evain-Brion D. Alterations of human placental epidermal growth factor receptor in intrauterine growth retardation. *J Clin Invest* (1994) 93:1149-1155. doi: 10.1172/JCI117067.

Fong G-H, Rossant J, Gertsenstein M, and Breitman ML. Role of the Flt-1 receptor tyrosine kinase in regulating the assembly of vascular endothelium. *Nature* (1995) 376:66-70.

Gabriel HD, Jung D, Butzler C, Temme A, Traub O, Winterhager E, et al. Transplacental uptake of glucose is decreased in embryonic lethal connexin26-deficient mice. *J. Cell Biol.* (1998) 140:1453-1461.

Giroux S, Tremblay M, Bernard D, Cardin-Girard JF, Aubry S, Larouche L, et al. Embryonic death of Mek1-deficient mice reveals a role for this kinase in angiogenesis in the labyrinthine region of the placenta. *Curr Biol* (1999) 9:369-372.

Guillemot F, Nagy A, Auerbach A, Rossant J, and Joyner AL. Essential role of Mash-2 in extraembryonic development. *Nature* (1994) 371:333-336. doi: 10.1038/371333a0.

Gurtner GC, Davis V, Li H, McCoy MJ, Sharpe A, and Cybulsky MI. Targeted disruption of the murine VCAM1 gene: essential role of VCAM-1 in chorioallantoic fusion and placentation. *Genes Dev* (1995) 9:1-14.

Hamada Y, Hiroe T, Suzuki Y, Oda M, Tsujimoto Y, Coleman JR, et al. Notch2 is required for formation of the placental circulatory system, but not for cell-type specification in the developing mouse placenta. *Differentiation* (2007) 75:268-278. doi: 10.1111/j.1432-0436.2006.00137.x.

Harbers K, Muller U, Grams A, Li E, Jaenisch R, and Franz T. Provirus integration into a gene encoding a ubiquitin-conjugating enzyme results in a placental defect and embryonic lethality. *Proc. Natl. Acad. Sci. U.S.A.* (1996) 93:12412-12417.

Hasan MZ, Ikawati M, Tocharus J, Kawaichi M, and Oka C. Abnormal development of placenta in HtrA1-deficient mice. *Dev Biol* (2015) 397:89-102. doi: 10.1016/j.ydbio.2014.10.015.

Hatano N, Mori Y, Oh-hora M, Kosugi A, Fujikawa T, Nakai N, et al. Essential role for ERK2 mitogen-activated protein kinase in placental development. *Genes Cells* (2003) 8:847-856.

Hildebrand JD, and Soriano P. Overlapping and unique roles for C-terminal binding protein 1 (CtBP1) and CtBP2 during mouse development. *Mol Cell Biol* (2002) 22:5296-5307.

Holdsworth-Carson SJ, Lim R, Mitton A, Whitehead C, Rice GE, Permezel M, et al. Peroxisome proliferator-activated receptors are altered in pathologies of the human placenta: gestational diabetes mellitus, intrauterine growth restriction and preeclampsia. *Placenta* (2010) 31:222-229. doi: 10.1016/j.placenta.2009.12.009.

Holdsworth-Carson SJ, Permezel M, Riley C, Rice GE, and Lappas M. Peroxisome proliferator-activated receptors and retinoid X receptor-alpha in term human gestational tissues: tissue specific and labour-associated changes. *Placenta* (2009) 30:176-186. doi: 10.1016/j.placenta.2008.11.013.

Huser M, Luckett J, Chiloeches A, Mercer K, Iwobi M, Giblett S, et al. MEK kinase activity is not necessary for Raf-1 function. *EMBO J* (2001) 20:1940-1951. doi: 10.1093/emboj/20.8.1940.

Itoh M, Yoshida Y, Nishida K, Narimatsu M, Hibi M, and Hirano T. Role of Gab1 in heart, placenta, and skin development and growth factor- and cytokine-induced extracellular signal-regulated kinase mitogen- activated protein kinase activation. *Mol Cell Biol* (2000) 20:3695-3704.

Kang MC, Park SJ, Kim HJ, Lee J, Yu DH, Bae KB, et al. Gestational loss and growth restriction by angiogenic defects in placental growth factor transgenic mice. *Arterioscler Thromb Vasc Biol* (2014) 34:2276-2282. doi: 10.1161/ATVBAHA.114.303693.

Kashif M, Hellwig A, Kolleker A, Shahzad K, Wang H, Lang S, et al. p45NF-E2 represses Gcm1 in trophoblast cells to regulate syncytium formation, placental vascularization and embryonic growth. *Development* (2011) 138:2235-2247. doi: 10.1242/dev.059105.

Kohli S, Hoffmann J, Lochmann F, Markmeyer P, Huebner H, Fahlbusch FB, et al. p45 NF-E2 regulates syncytiotrophoblast differentiation by post-translational GCM1 modifications in human intrauterine growth restriction. *Cell Death Dis* (2017) 8:e2730. doi: 10.1038/cddis.2017.127.

Kozak KR, Abbott B, and Hankinson O. ARNT-deficient mice and placental differentiation. *Dev Biol* (1997) 191:297-305. doi: 10.1006/dbio.1997.8758.

Krebs LT, Shutter JR, Tanigaki K, Honjo T, Stark KL, and Gridley T. Haploinsufficient lethality and formation of arteriovenous malformations in Notch pathway mutants. *Genes Dev* (2004) 18:2469-2473. doi: 10.1101/gad.1239204.

Krebs LT, Xue Y, Norton CR, Shutter JR, Maguire M, Sundberg JP, et al. Notch signaling is essential for vascular morphogenesis in mice. *Genes Dev* (2000) 14:1343-1352.

Kruger O, Plum A, Kim J, Winterhager E, Maxeiner S, Hallas G, et al. Defective vascular development in connexin 45-deficient mice. *Development* (2000) 127:4179-4193.

Kuang SQ, Liao L, Zhang H, Pereira FA, Yuan Y, DeMayo FJ, et al. Deletion of the cancer-amplified coactivator AIB3 results in defective placentation and embryonic lethality. *J Biol Chem* (2002) 277:45356-45360. doi: 10.1074/jbc.C200509200.

Kuhnel E, Kleff V, Stojanovska V, Kaiser S, Waldschutz R, Herse F, et al. Placental-Specific Overexpression of sFlt-1 Alters Trophoblast Differentiation and Nutrient Transporter Expression in an IUGR Mouse Model. *J Cell Biochem* (2017) 118:1316-1329. doi: 10.1002/jcb.25789.

Kumar N, Leverence J, Bick D, and Sampath V. Ontogeny of growth-regulating genes in the placenta. *Placenta* (2012) 33:94-99. doi: 10.1016/j.placenta.2011.11.018.

Kwee L, Baldwin HS, Shen HM, Stewart CL, Buck C, Buck CA, et al. Defective development of the embryonic and extraembryonic circulatory systems in vascular cell adhesion molecule (VCAM-1) deficient mice. *Development* (1995) 121:489-503.

Kweider N, Huppertz B, Rath W, Lambertz J, Caspers R, ElMoursi M, et al. The effects of Nrf2 deletion on placental morphology and exchange capacity in the mouse. *J Matern Fetal Neonatal Med* (2017) 30:2068-2073. doi: 10.1080/14767058.2016.1236251.

Lacko LA, Hurtado R, Hinds S, Poulos MG, Butler JM, and Stuhlmann H. Altered feto-placental vascularization, feto-placental malperfusion and fetal growth restriction in mice with Egfl7 loss of function. *Development* (2017) 144:2469-2479. doi: 10.1242/dev.147025.

Laviola L, Perrini S, Belsanti G, Natalicchio A, Montrone C, Leonardini A, et al. Intrauterine growth restriction in humans is associated with abnormalities in placental insulin-like growth factor signaling. *Endocrinology* (2005) 146:1498-1505. doi: 10.1210/en.2004-1332.

Li G, Xu C, Lin X, Qu L, Xia D, Hongdu B, et al. Deletion of Pdcd5 in mice led to the deficiency of placenta development and embryonic lethality. *Cell Death Dis* (2017) 8:e2811. doi: 10.1038/cddis.2017.124.

Li Y, and Behringer RR. Esx1 is an X-chromosome-imprinted regulator of placental development and fetal growth. *Nat. Genet.* (1998) 20:309-311.

Lotz K, Pyrowolakis G, and Jentsch S. BRUCE, a giant E2/E3 ubiquitin ligase and inhibitor of apoptosis protein of the trans-Golgi network, is required for normal placenta development and mouse survival. *Mol Cell Biol* (2004) 24:9339-9350. doi: 10.1128/MCB.24.21.9339-9350.2004.

Lyall F, Young A, Boswell F, Kingdom JC, and Greer IA. Placental expression of vascular endothelial growth factor in placentae from pregnancies complicated by pre-eclampsia and intrauterine growth restriction does not support placental hypoxia at delivery. *Placenta* (1997) 18:269-276.

Ma GT, Soloveva V, Tzeng SJ, Lowe LA, Pfendler KC, Iannaccone PM, et al. Nodal regulates trophoblast differentiation and placental development. *Dev Biol* (2001) 236:124-135. doi: 10.1006/dbio.2001.0334.

Ma S, Charron J, and Erikson RL. Role of Plk2 (Snk) in mouse development and cell proliferation. *Mol Cell Biol* (2003) 23:6936-6943.

McMinn J, Wei M, Schupf N, Cusmai J, Johnson EB, Smith AC, et al. Unbalanced placental expression of imprinted genes in human intrauterine growth restriction. *Placenta* (2006) 27:540-549. doi: 10.1016/j.placenta.2005.07.004.

Miner JH, Cunningham J, and Sanes JR. Roles for laminin in embryogenesis: exencephaly, syndactyly, and placentopathy in mice lacking the laminin alpha5 chain. *J Cell Biol* (1998) 143:1713-1723.

Mo FE, Muntean AG, Chen CC, Stolz DB, Watkins SC, and Lau LF. CYR61 (CCN1) is essential for placental development and vascular integrity. *Mol Cell Biol* (2002) 22:8709-8720.

Monkley SJ, Delaney SJ, Pennisi DJ, Christiansen JH, and Wainwright BJ. Targeted disruption of the Wnt2 gene results in placentation defects. *Development* (1996) 122:3343-3353.

Morasso MI, Grinberg A, Robinson G, Sargent TD, and Mahon KA. Placental failure in mice lacking the homeobox gene Dlx3. *Proc. Natl. Acad. Sci. U.S.A.* (1999) 96:162-167.

Moser M, Li Y, Vaupel K, Kretzschmar D, Kluge R, Glynn P, et al. Placental failure and impaired vasculogenesis result in embryonic lethality for neuropathy target esterase-deficient mice. *Mol Cell Biol* (2004) 24:1667-1679.

Mudgett JS, Ding J, Guh-Siesel L, Chartrain NA, Yang L, Gopal S, et al. Essential role for p38alpha mitogen-activated protein kinase in placental angiogenesis. *Proc Natl Acad Sci U S A* (2000) 97:10454-10459.

Murthi P, Doherty VL, Said JM, Donath S, Brennecke SP, and Kalionis B. Homeobox gene ESX1L expression is decreased in human pre-term idiopathic fetal growth restriction. *Mol Hum Reprod* (2006) 12:335-340. doi: 10.1093/molehr/gal037.

Nadeau V, and Charron J. Essential role of the ERK/MAPK pathway in blood-placental barrier formation. *Development* (2014) 141:2825-2837. doi: 10.1242/dev.107409.

Nevo O, Many A, Xu J, Kingdom J, Piccoli E, Zamudio S, et al. Placental expression of soluble fms-like tyrosine kinase 1 is increased in singletons and twin pregnancies with intrauterine growth restriction. *J Clin Endocrinol Metab* (2008) 93:285-292. doi: 10.1210/jc.2007-1042.

Oh-McGinnis R, Bogutz AB, and Lefebvre L. Partial loss of Ascl2 function affects all three layers of the mature placenta and causes intrauterine growth restriction. *Dev Biol* (2011) 351:277-286. doi: 10.1016/j.ydbio.2011.01.008.

Ohlsson R, Falck P, Hellstrom M, Lindahl P, Bostrom H, Franklin G, et al. PDGFB regulates the development of the labyrinthine layer of the mouse fetal placenta. *Dev Biol* (1999) 212:124-136.

Parast MM, Yu H, Ciric A, Salata MW, Davis V, and Milstone DS. PPARgamma regulates trophoblast proliferation and promotes labyrinthine trilineage differentiation. *PLoS One* (2009) 4:e8055. doi: 10.1371/journal.pone.0008055.

Parekh V, McEwen A, Barbour V, Takahashi Y, Rehg JE, Jane SM, et al. Defective extraembryonic angiogenesis in mice lacking LBP-1a, a member of the grainyhead family of transcription factors. *Mol Cell Biol* (2004) 24:7113-7129. doi: 10.1128/MCB.24.16.7113-7129.2004.

Perez-Garcia V, Fineberg E, Wilson R, Murray A, Mazzeo CI, Tudor C, et al. Placentation defects are highly prevalent in embryonic lethal mouse mutants. *Nature* (2018) 555:463-468. doi: 10.1038/nature26002.

Qian X, Esteban L, Vass WC, Upadhyaya C, Papageorge AG, Yienger K, et al. The Sos1 and Sos2 Ras-specific exchange factors: differences in placental expression and signaling properties. *Embo J* (2000) 19:642-654.

Rajaraman G, Murthi P, Pathirage N, Brennecke SP, and Kalionis B. Downstream targets of homeobox gene HLX show altered expression in human idiopathic fetal growth restriction. *Am J Pathol* (2010) 176:278-287. doi: 10.2353/ajpath.2010.090187.

Rampon C, Bouillot S, Climescu-Haulica A, Prandini MH, Cand F, Vandenbrouck Y, et al. Protocadherin 12 deficiency alters morphogenesis and transcriptional profile of the placenta. *Physiol Genomics* (2008) 34:193-204. doi: 10.1152/physiolgenomics.00220.2007.

Rodriguez TA, Sparrow DB, Scott AN, Withington SL, Preis JI, Michalicek J, et al. Cited1 is required in trophoblasts for placental development and for embryo growth and survival. *Mol Cell Biol* (2004) 24:228-244.

Sahin Z, Acar N, Ozbey O, Ustunel I, and Demir R. Distribution of Notch family proteins in intrauterine growth restriction and hypertension complicated human term placentas. *Acta Histochem* (2011) 113:270-276. doi: 10.1016/j.acthis.2009.10.006.

Sarkar AA, Nuwayhid SJ, Maynard T, Ghandchi F, Hill JT, Lamantia AS, et al. Hectd1 is required for development of the junctional zone of the placenta. *Dev Biol* (2014) 392:368-380. doi: 10.1016/j.ydbio.2014.05.007.

Sarkar AA, Sabatino JA, Sugrue KF, and Zohn IE. Abnormal labyrinthine zone in the Hectd1-null placenta. *Placenta* (2016) 38:16-23. doi: 10.1016/j.placenta.2015.12.002.

Saxton TM, Cheng AM, Ong SH, Lu Y, Sakai R, Cross JC, et al. Gene dosage-dependent functions for phosphotyrosine-Grb2 signaling during mammalian tissue morphogenesis. *Curr Biol* (2001) 11:662-670.

Schmidt C, Bladt F, Goedecke S, Brinkmann V, Zschiesche W, Sharpe M, et al. Scatter factor/hepatocyte growth factor is essential for liver development. *Nature* (1995) 373:699-702.

Schorpp-Kistner M, Wang ZQ, Angel P, and Wagner EF. JunB is essential for mammalian placentation. *EMBO J.* (1999) 18:934-948.

Schreiber M, Wang ZQ, Jochum W, Fetka I, Elliott C, and Wagner EF. Placental vascularisation requires the AP-1 component fra1. *Development* (2000) 127:4937-4948.

Sharma N, Kubaczka C, Kaiser S, Nettersheim D, Mughal SS, Riesenberg S, et al. Tpbpa-Cre-mediated deletion of TFAP2C leads to deregulation of Cdkn1a, Akt1 and the ERK pathway, causing placental growth arrest. *Development* (2016) 143:787-798. doi: 10.1242/dev.128553.

Shi W, van den Hurk JA, Alamo-Bethencourt V, Mayer W, Winkens HJ, Ropers HH, et al. Choroideremia gene product affects trophoblast development and vascularization in mouse extra-embryonic tissues. *Dev Biol* (2004) 272:53-65.

Sibilia M, and Wagner EF. Strain-dependent epithelial defects in mice lacking the EGF receptor. *Science* (1995) 269:234-238.

Sibley CP, Coan PM, Ferguson-Smith AC, Dean W, Hughes J, Smith P, et al. Placental-specific insulin-like growth factor 2 (Igf2) regulates the diffusional exchange characteristics of the mouse placenta. *Proc Natl Acad Sci U S A* (2004) 101:8204-8208.

Sibley CP, Pardi G, Cetin I, Todros T, Piccoli E, Kaufmann P, et al. Pathogenesis of intrauterine growth restriction (IUGR)-conclusions derived from a European Union Biomed 2 Concerted Action project 'Importance of Oxygen Supply in Intrauterine Growth Restricted Pregnancies'-a workshop report. *Placenta* (2002) 23 Suppl A:S75-79. doi: 10.1053/plac.2002.0796.

Sohn SJ, Sarvis BK, Cado D, and Winoto A. ERK5 MAPK regulates embryonic angiogenesis and acts as a hypoxia-sensitive repressor of vascular endothelial growth factor expression. *J Biol Chem* (2002) 277:43344-43351. doi: 10.1074/jbc.M207573200.

Somerset DA, Li XF, Afford S, Strain AJ, Ahmed A, Sangha RK, et al. Ontogeny of hepatocyte growth factor (HGF) and its receptor (c-met) in human placenta: reduced HGF expression in intrauterine growth restriction. *Am J Pathol* (1998) 153:1139-1147. doi: 10.1016/S0002-9440(10)65658-1.

Steingrimsson E, Tessarollo L, Reid SW, Jenkins NA, and Copeland NG. The bHLH-Zip transcription factor Tfeb is essential for placental vascularization. *Development* (1998) 125:4607-4616.

Stumpo DJ, Byrd NA, Phillips RS, Ghosh S, Maronpot RR, Castranio T, et al. Chorioallantoic fusion defects and embryonic lethality resulting from disruption of Zfp36L1, a gene encoding a CCCH tandem zinc finger protein of the Tristetraprolin family. *Mol Cell Biol* (2004) 24:6445-6455. doi: 10.1128/MCB.24.14.6445-6455.2004.

Szentpeteri I, Rab A, Kornya L, Kovacs P, and Joo JG. Gene expression patterns of vascular endothelial growth factor (VEGF-A) in human placenta from pregnancies with intrauterine growth restriction. *J Matern Fetal Neonatal Med* (2013) 26:984-989. doi: 10.3109/14767058.2013.766702.

Tamai Y, Ishikawa T, Bosl MR, Mori M, Nozaki M, Baribault H, et al. Cytokeratins 8 and 19 in the mouse placental development. *J Cell Biol* (2000) 151:563-572.

Tetzlaff MT, Bai C, Finegold M, Wilson J, Harper JW, Mahon KA, et al. Cyclin F disruption compromises placental development and affects normal cell cycle execution. *Mol Cell Biol* (2004a) 24:2487-2498.

Tetzlaff MT, Yu W, Li M, Zhang P, Finegold M, Mahon K, et al. Defective cardiovascular development and elevated cyclin E and Notch proteins in mice lacking the Fbw7 F-box protein. *Proc Natl Acad Sci U S A* (2004b) 101:3338-3345. doi: 10.1073/pnas.0307875101.

Threadgill DW, Dlugosz AA, Hansen LA, Tennenbaum T, Lichti U, Yee D, et al. Targeted disruption of mouse EGF receptor: effect of genetic background on mutant phenotype. *Science* (1995) 269:230-234.

Thumkeo D, Keel J, Ishizaki T, Hirose M, Nonomura K, Oshima H, et al. Targeted disruption of the mouse rho-associated kinase 2 gene results in intrauterine growth retardation and fetal death. *Mol Cell Biol* (2003) 23:5043-5055.

Tian Y, Jackson P, Gunter C, Wang J, Rock CO, and Jackowski S. Placental thrombosis and spontaneous fetal death in mice deficient in ethanolamine kinase 2. *J Biol Chem* (2006) 281:28438-28449. doi: 10.1074/jbc.M605861200.

Tong MH, Jiang H, Liu P, Lawson JA, Brass LF, and Song WC. Spontaneous fetal loss caused by placental thrombosis in estrogen sulfotransferase-deficient mice. *Nat Med* (2005) 11:153-159. doi: 10.1038/nm1184.

Tunster SJ, Creeth HDJ, and John RM. The imprinted Phlda2 gene modulates a major endocrine compartment of the placenta to regulate placental demands for maternal resources. *Dev Biol* (2016a) 409:251-260. doi: 10.1016/j.ydbio.2015.10.015.

Tunster SJ, McNamara GI, Creeth HDJ, and John RM. Increased dosage of the imprinted Ascl2 gene restrains two key endocrine lineages of the mouse Placenta. *Dev Biol* (2016b) 418:55-65. doi: 10.1016/j.ydbio.2016.08.014.

Tunster SJ, Tycko B, and John RM. The imprinted Phlda2 gene regulates extraembryonic energy stores. *Mol Cell Biol* (2010) 30:295-306. doi: 10.1128/MCB.00662-09.

Uehara Y, Minowa O, Mori C, Shiota K, Kuno J, Noda T, et al. Placental defect and embryonic lethality in mice lacking hepatocyte growth factor/scatter factor. *Nature* (1995) 373:702-705.

Ueno M, Lee LK, Chhabra A, Kim YJ, Sasidharan R, Van Handel B, et al. c-Met-dependent multipotent labyrinth trophoblast progenitors establish placental exchange interface. *Dev Cell* (2013) 27:373-386. doi: 10.1016/j.devcel.2013.10.019.

Ware CB, Horowitz MC, Renshaw BR, Hunt JS, Liggitt D, Koblar SA, et al. Targeted disruption of the low-affinity leukemia inhibitory factor receptor gene causes placental, skeletal, neural and metabolic defects and results in perinatal death. *Development* (1995) 121:1283-1299.

Wu L, de Bruin A, Saavedra HI, Starovic M, Trimboli A, Yang Y, et al. Extra-embryonic function of Rb is essential for embryonic development and viability. *Nature* (2003) 421:942-947. doi: 10.1038/nature01417.

Xiao X, Zhao Y, Jin R, Chen J, Wang X, Baccarelli A, et al. Fetal growth restriction and methylation of growth-related genes in the placenta. *Epigenomics* (2016) 8:33-42. doi: 10.2217/epi.15.101.

Xu X, Weinstein M, Li C, Naski M, Cohen RI, Ornitz DM, et al. Fibroblast growth factor receptor 2 (FGFR2)-mediated reciprocal regulation loop between FGF8 and FGF10 is essential for limb induction. *Development* (1998) 125:753-765.

Yang J, Boerm M, McCarty M, Bucana C, Fidler IJ, Zhuang Y, et al. Mekk3 is essential for early embryonic cardiovascular development. *Nat Genet* (2000) 24:309-313. doi: 10.1038/73550.

Yang JT, Rayburn H, and Hynes RO. Cell adhesion events mediated by alpha 4 integrins are essential in placental and cardiac development. *Development* (1995) 121:549-560.

Yang ZZ, Tschopp O, Hemmings-Mieszczak M, Feng J, Brodbeck D, Perentes E, et al. Protein kinase B alpha/Akt1 regulates placental development and fetal growth. *J Biol Chem* (2003) 278:32124-32131. doi: 10.1074/jbc.M302847200.

Ylikorkala A, Rossi DJ, Korsisaari N, Luukko K, Alitalo K, Henkemeyer M, et al. Vascular abnormalities and deregulation of VEGF in Lkb1-deficient mice. *Science* (2001) 293:1323-1326. doi: 10.1126/science.1062074.

Yung HW, Hemberger M, Watson ED, Senner CE, Jones CP, Kaufman RJ, et al. Endoplasmic reticulum stress disrupts placental morphogenesis: implications for human intrauterine growth restriction. *Journal of Pathology* (2012). doi: 10.1002/path.4068.

Zhu J, Motejlek K, Wang D, Zang K, Schmidt A, and Reichardt LF. Beta8 integrins are required for vascular morphogenesis in mouse embryos. *Development* (2002) 129:2891-2903.

Zygmunt M, Boving B, Wienhard J, Munstedt K, Braems G, Bohle RM, et al. Expression of cell adhesion molecules in the extravillous trophoblast is altered in IUGR. *Am J Reprod Immunol* (1997) 38:295-301.
